# The Impact of Microfibril Orientations on the Biomechanics of Plant Cell Walls and Tissues

**DOI:** 10.1007/s11538-016-0207-8

**Published:** 2016-10-19

**Authors:** Mariya Ptashnyk, Brian Seguin

**Affiliations:** 1 Division of Mathematics, University of Dundee, Dundee, DD1 4HN UK; 2 Department of Mathematics and Statistics, Loyola University Chicago, Chicago, IL 60660 USA

**Keywords:** Biomechanics, Plant modelling, Homogenization, Linear elasticity, Plant cell wall microfibrils

## Abstract

The microscopic structure and anisotropy of plant cell walls greatly influence the mechanical properties, morphogenesis, and growth of plant cells and tissues. The microscopic structure and properties of cell walls are determined by the orientation and mechanical properties of the cellulose microfibrils and the mechanical properties of the cell wall matrix. Viewing the shape of a plant cell as a square prism with the axis aligning with the primary direction of expansion and growth, the orientation of the microfibrils within the side walls, i.e. the parts of the cell walls on the sides of the cells, is known. However, not much is known about their orientation at the upper and lower ends of the cell. Here we investigate the impact of the orientation of cellulose microfibrils within the upper and lower parts of the plant cell walls by solving the equations of linear elasticity numerically. Three different scenarios for the orientation of the microfibrils are considered. We also distinguish between the microstructure in the side walls given by microfibrils perpendicular to the main direction of the expansion and the situation where the microfibrils are rotated through the wall thickness. The macroscopic elastic properties of the cell wall are obtained using homogenization theory from the microscopic description of the elastic properties of the cell wall microfibrils and wall matrix. It is found that the orientation of the microfibrils in the upper and lower parts of the cell walls affects the expansion of the cell in the lateral directions and is particularly important in the case of forces acting on plant cell walls and tissues.

## Introduction

To better understand plant development, it is important to analyse how the microscopic structure of plant tissues and organs impacts their mechanical properties. The elastic properties of plant tissues are strongly determined by the mechanical properties of the cell walls surrounding plant cells and by the cross-linked pectin network of the middle lamella which joins individual cells together. Primary cell walls of plant cells consist mainly of oriented cellulose microfibrils, pectin, hemicellulose, proteins, and water. The orientation, length, and high tensile strength of the microfibrils strongly influence the wall’s stiffness. Hemicelluloses form hydrogen bonds with the surface of cellulose microfibrils, which may affect the mechanical strength of the cell wall by creating a microfibril–hemicellulose network (Somerville et al. 2004). Pectin, once it is de-esterified and cross-linked with calcium ions, forms a gel within the primary cell wall and middle lamella and is hypothesized to be one of the main regulators of cell wall elasticity (Wolf et al. 2012).

Since the turgor pressure acts isotropically, it is the microstructure of the cell walls, e.g. the orientation of the cellulose microfibrils, which determines the anisotropic deformation and expansion of plant cells. Many plant cells, especially cells in plant roots and stem tissues, have a primary direction of expansion and less expansion takes place in the directions orthogonal to it; see, e.g. Green (1965) and Probine and Preston (1962). It is well known that cellulose microfibrils are parallel to the sides of primary cell walls and, particularly in young cells, perpendicular to the main direction of extension and growth (Green 1999; MacKinnon et al. 2006; Sugimato et al. 2000; Szymanski and Cosgrove 2009). While the cells are elongating, the microfibrils may reorientate in the main direction of growth (Anderson et al. 2010). For plant cells whose shape can be approximated by a prism or cylinder with the axis aligned with the primary direction of expansion, the microfibrils within the cell walls making up the sides of the cell are parallel to the sides and perpendicular to the axis of the cell or create a plywood-like microstructure of rotated layers of microfibrils. However, the orientation of the microfibrils in the upper and lower parts of plant cells does not appear to be known, possibly due to restrictions in imaging these parts of the cell walls.

Due to the impact of cell wall anisotropy on plant tissue biomechanics and the importance of multiscale modelling of plant biomechanics (Baskin 2005; Baskin and Jensen 2013; Jensen and Fozard 2015), knowing the orientation of the microfibrils in each part of the plant cell walls and the qualitative and quantitative impact of the microfibrils orientation and distribution on the mechanical properties of cell walls and tissues is essential.

Different modelling approaches have been used to account for the impact of the microstructure and anisotropy of plant cell walls on the mechanical properties and growth of plant cells and tissues. In Veytsman and Cosgrove (1998), the microstructure of cell walls was addressed by distinguishing between the free energies related to the elasticity of macromolecules and hydrogen bonds, respectively. The theory of anisotropic visco-plasticity and a thin shell model were used in Dumais et al. (2006) to describe the anisotropic growth of a tip of a plant cell. The “decomposition approach” (the splitting of the deformation tensor into an elastic and a growth part) has been applied to model the growth of a part of a cell wall and its hardening due to changes in the chemical properties of the cell wall matrix (Huang et al. 2012). Here the impact of microfibrils aligned in the direction orthogonal to the main direction of expansion was addressed phenomenologically in the free energy function. The impact of the dynamics of hemicellulose cross-links on the growth of a plant cell wall was analysed in Dyson et al. (2012). It was shown using a mathematical model for hemicellulose cross-link dynamics that the strain-induced cross-link breakages influence the yield stress, whereas enzymes soften the wall in its pre-yield state. In Dyson and Jensen (2010), the influence of microfibril orientation and the external torque on the expansion process was analysed by representing the primary cell wall as a thin axisymmetric fibre-reinforced viscous sheet and assuming that fibres are stretched and reoriented by the flow. A vertex-element model for plant tissue deformation and growth was considered in Fozard et al. (2013). The impact of microfibrils on the mechanical properties of cell walls was accounted for by introducing an anisotropic viscous stress which depends on a pair of microfibril directions.

In previous works, the influence of the anisotropic microstructure and of the orientation of microfibrils on the mechanical properties of plant cell walls was considered by including the orientation of the microfibrils in the expression of the stress tensor in a phenomenological way. In this work we consider the microscopic structure of the cell walls explicitly and define a microscopic model for the elastic deformations of plant cell walls and tissues on the scale of the microfibrils. In such a way we can consider different orientations of microfibrils explicitly and distinguish between the mechanical properties of the microfibrils and the cell wall matrix. Using multiscale analysis techniques, we rigorously derive macroscopic properties for the cell wall from a microscopic description and analyse the impact of the microscopic structure on the elastic deformations of cells in a more detailed way. Our model also accounts for the distribution of forces between the cells.

In this paper we investigate the impact of the orientation of the cellulose microfibrils in the upper and lower parts of cell walls and of a rotated (plywood-like) distribution of microfibrils in the side walls on the elastic deformation of the plant cell walls and tissues using multiscale modelling and numerical simulations. Modelling plant cells as square prisms with rounded edges, we consider a part of a plant tissue represented by a “central” cell surrounded by cells on all sides. The cell walls and middle lamella are modelled as linearly elastic materials, and on the internal boundaries we specify traction boundary conditions to represent the turgor pressure. Within the sides of the cell walls, we consider the cases where the cellulose microfibrils are either arranged periodically, see, e.g. Thomas et al. (2013), or are arranged so that their orientation changes through the thickness of the cell wall; see, e.g. Anderson et al. (2010). The length scale of the microfibrils (their diameter and separation distance between microfibrils) is much smaller than the length scale associated with the thickness of the cell wall. This smaller length scale will be referred to as the microscale, while the scale associated with the dimensions of the cell wall is called the macroscale. To obtain the elastic properties of the primary cell wall, we follow (Ptashnyk and Seguin 2016a) and use techniques of periodic homogenization to determine a macroscopic (effective) elasticity tensor that depends on the orientation of the microfibrils on the microscale. It was observed experimentally that calcium–pectin cross-links influence the mechanical properties of the cell wall matrix and middle lamella, e.g. Wolf et al. (2012). The impact of the density of the calcium–pectin cross-links on the elastic properties of the cell walls is modelled through the Young’s modulus of the isotropic cell wall matrix. Since it is known that the microfibrils are not isotropic (Diddens et al. 2008), they are assumed to be transversely isotropic. The macroscopic (effective) elasticity tensor for the cell walls is determined from the microscopic description of the mechanical properties of the microfibrils and cell wall matrix by solving numerically the corresponding problems defined on a Representative Volume Element (RVE), which reflect the underlying microscopic structure of the cell walls. Then, using the macroscopic elasticity tensor for different microfibril orientations, we solve numerically the equations of linear elasticity in a domain corresponding to a part of a plant tissue with different traction boundary conditions. The effect of a shift in the position of neighbouring cells relative to each other on the elastic deformations of a plant tissue is analysed by considered two different configurations: one with cells shifted relative to each other and another where the cells are distributed symmetrically without a shift in their position relative to each other. The impact of the microfibrils reorientation, observed experimentally in Anderson et al. (2010), is analysed by considering the spatially dependent rotation of the effective elasticity tensor. In this article we consider elastic deformations of plant cell walls and tissues. Using the “decomposition approach” and splitting the deformation gradient into an elastic and growth part, it is possible that these results can be extended to analyse the interactions between the microstructure of plant cell walls and growth.

We find that different configurations of orientations of microfibrils in the upper and lower parts of the cell walls do have an impact on the elastic deformation of the plant cell walls and tissues in the directions parallel to the upper and lower parts of the cell walls and have little effect on the expansion of the cells in the direction of their axes. For a plant tissue with a staggered distribution of cells, the arrangement of the microfibrils in the upper and lower parts of the cell walls has an impact on the elastic deformation also in the absence of external forces, in contrast to a plant tissue without a shift in the positions of cells relative to the neighbouring cells. In the case where the microstructure in the side walls of the cells is defined by layers of microfibrils rotated through the wall thickness, we obtain a much smaller expansion in the direction of the cell’s axis and the orientation of the microfibrils in the upper and lower parts impacts the deformation in the directions orthogonal to the cell’s axis. We also observe that for a plant tissue without a shift in the positions of the cells relative to the neighbouring cells, the maximal displacements in the directions orthogonal to the cell’s axis are smaller than in the case of a staggered distribution of cells. We also find that the expansion of the cell in the direction of its axis is smaller for shorter cells, which is in accord with Hooke’s law. The difference in the values of the turgor pressure in the neighbouring cells causes larger deformations in the directions parallel to the upper and lower parts of the cell walls.

The outline of the paper is as follows. In Sect. 2, we specify our model for plant tissue biomechanics. We consider the elastic deformation of the primary cell walls joined by middle lamella and the cell inside is modelled by prescribing a turgor pressure. Next, in Sect. 3, the results of numerical simulations and a discussion of simulation results are presented. Concluding discussions are presented in Sect. 4.

## Statement of the Mathematical Model for Plant Tissue Biomechanics

We start by presenting our model for the elastic deformations of a part of a plant tissue. This section is divided into three parts: a description of the geometry of the domain, the presentation of the governing equations and boundary conditions, and the specification of the elasticity tensor in the domains representing the different parts of plant cell walls and middle lamella.

### Geometry

Our geometry is motivated by the structure of cells and tissues in young plant roots. Based on representative values from scanned images of plant root cells in the elongation zone (private communication), we assume that the length of a cell is $37.2 \, \upmu$m and the width of a cell is $17.92\, \upmu$m. The range for a typical wall thickness is 0.1–2 $\upmu$m; see, e.g. Dumais et al. (2006), Niklas (1992), and in our model we consider the cell wall thickness to be equal to $1 \,\upmu$m. We assume that the thickness of the middle lamella is approximately 1 / 5 of the thickness of the cell wall, so that the thickness of the middle lamella is taken to be $0.2 \,\upmu$m.

**Fig. 1:**
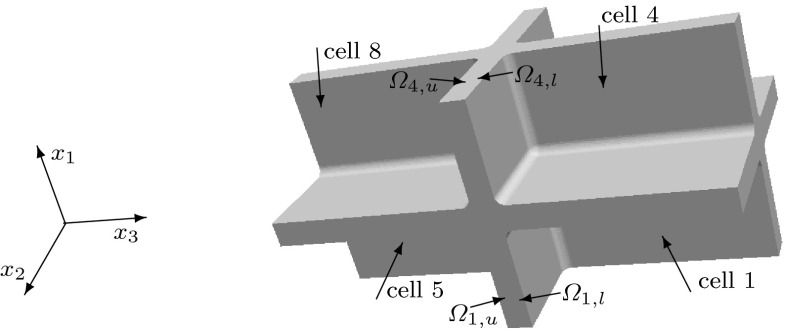
The domain $\Omega$ consisting of parts of eight cells without a shift in the position of neighbouring cells; the cell length is $37.2\, \upmu$m and the cell width is $17.92\, \upmu$m; $\Omega _{j,u}$ and $\Omega _{j,l}$ denote the upper and lower parts of the cell walls in subdomains $\Omega _j$, with $j=1,2,3,4$, respectively

**Fig. 2:**
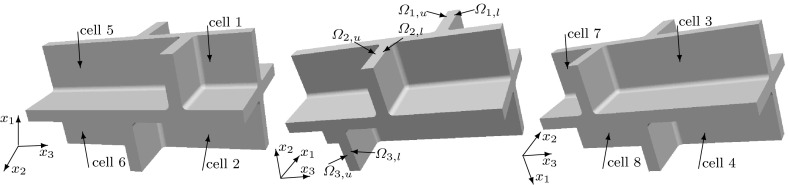
The domain $\Omega$ consisting of parts of eight cells with two pairs of diagonally opposite cells having the same position on the $x_3$-axis and the four other cells are shifted by $9.3\, \upmu$m relative to the neighbouring cells; the cell length is $37.2\, \upmu$m and the cell width is $17.92\, \upmu$m; $\Omega _{j,u}$ and $\Omega _{j,l}$ denote the upper and lower parts of the cell walls in subdomains $\Omega _j$, with $j=1,2,3,4$, respectively. See also Fig. 5

We consider a domain composed of parts of eight cells connected by the middle lamella, where the shape of a plant cell is approximated by a square prism with rounded edges. The $(x_1,x_2,x_3)$ coordinate system is chosen so that the origin is inside of a cell with the axes parallel to the edges of the prism of this cell and the $x_3$-axis is aligned with the axis of the cell. We consider the domain $\Omega$, the bounding box of which is $(0, x_{1,\mathrm{max}})\times (0, x_{2,\mathrm{max}})\times (0, x_{3,\mathrm{max}})$, where $x_{1,\mathrm {max}}= 20.12$, $x_{2,\mathrm {max}}= 20.12$, $x_{3,\mathrm {max}}= 39.4$. If a symmetric distribution of cells in a plant tissue is assumed, see Fig. 1, we obtain that $x_1=0$, $x_2=0$, and $x_3=0$ are planes of symmetry, and by reflecting $\Omega$ over the planes $x_1=0$, $x_2=0$, and $x_3=0$, we obtain a domain that includes a central cell and parts of the 26 cells that surround it. This motivates us to consider a domain $\Omega$ composed of parts of eight cells to represent a part of a plant tissue also in the case of a staggered distribution of cells. We assume that neighbouring cells are positioned shifted along the $x_3$-axis relative to each other with a shift of 1/4 of the cell wall length, i.e. a shift of $9.3\, \upmu$m; see Fig. 2. In this case, we only have symmetries across the planes $x_1=0$ and $x_2=0$. A cross section of $\Omega$ at a constant value of $x_3$ in $(0,8.5)\cup (12.3,17.8)\cup (21.6,27.1)\cup (30.9,39.4)$ is shown in Fig. 4. The cross sections of $\Omega$ for a constant $x_3$-value in (9.3, 11.5), (18.6, 20.4) and (27.9, 30.1) are shown in Fig. 5. For other values of $x_3$, the cross sections have different shapes due to the rounded edges of the domain; see Fig. 2. A cross section of $\Omega$ at a constant value of $x_1$ in (0, 8.16) is shown in Fig. 6. A representation of cross sections of $\Omega$ at other values of $x_1$ or $x_2$ can be obtained by rotating or reflecting the geometry in Fig. 6 appropriately.

**Fig. 3:**
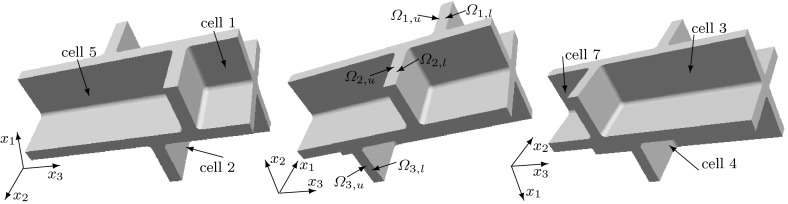
The domain $\Omega$ consisting of parts of eight cells where the position on the $x_3$-axis of each pair of upper and lower cells is shifted relative to a neighbouring pair of cells, in contrast to the domain $\Omega$ depicted in Fig. 2 where two diagonally opposite pairs of cells have the same positions on the $x_3$-axis; the cell length is $37.2\, \upmu$m, the cell width is $17.92\, \upmu$m, and the shift is $6.4\, \upmu$m$;\Omega _{j,u}$ and $\Omega _{j,l}$ denote the upper and lower parts of the cell walls in subdomains $\Omega _j$, with $j=1,2,3,4$, respectively

**Fig. 4:**
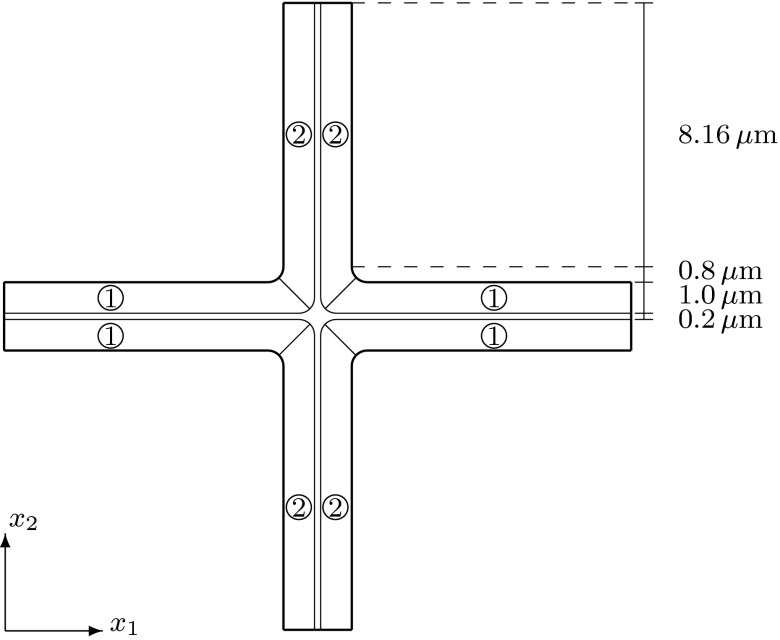
A cross section of $\Omega$ at a constant $x_3$-value in $(0,8.5)\cup (12.3,17.8)\cup (21.6,27.1)\cup (30.9,39.4)$. All rounded corners in this figure have a radius of $0.8 \, \upmu$m. The region that is not marked is the middle lamella, which has no microfibrils. The regions marked with 1 have cellulose microfibrils parallel to the $x_1$-axis or microfibrils rotated through the thickness of the cell wall and are parallel to the $x_1$-axis at the inner part of the cell wall and parallel to the $x_3$-axis near the middle lamella. The regions marked with 2 have microfibrils parallel to the $x_2$-axis or microfibrils rotated through the thickness of the cell wall and are parallel to the $x_2$-axis at the inner part of the cell wall and parallel to the $x_3$-axis near the middle lamella. This cross section is symmetric about the lines $x_1=10.06$, $x_2=10.06$, and $x_1=x_2$

**Fig. 5:**
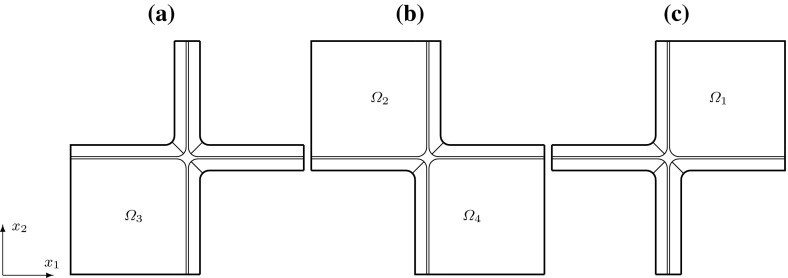
Cross sections of $\Omega$ for an $x_3$-value **a** in (9.3, 11.5), **b** in (18.6, 20.4), or **c** in (27.9, 30.1). Each of the square regions consists of an upper part of a cell wall $\Omega _{j,u}$ (smaller $x_3$-value) and a lower part of a cell wall $\Omega _{j,l}$ (larger $x_3$-value) separated by middle lamella, where $j=1,2,3,4$. The upper and lower parts of the cell walls are $1\, \upmu$m thick, and the middle lamella is $0.2\, \upmu$m thick. The regions with microfibrils, corresponding to the parts of the cell walls, have a length of $9.96\,\upmu$m on each side. All of the rounded corners have a radius of 0.8 $\upmu$m

We will label the eight cells in the domain $\Omega$ in the following way: the four upper cells we label from 1 to 4 by starting with the cell occupying the subdomain $(11.16 ,20.12)\times (11.16,20.12)\times (30.9,39.4)$, see Fig. 2, and proceeding counterclockwise. The cells below the cells 1, 2, 3, and 4 we label as 5, 6, 7, and 8, respectively; see Fig. 2. Notice that the origin (0, 0, 0) is located in cell 7.

We also consider the symmetric eight cells geometry without a shift in the position of cells, see Fig. 1, and the geometric configuration of the eight cells domain where each pair of upper and lower cells is shifted relative to a neighbouring pair of cells with a shift equal to $6.4\, \upmu$m; see Fig. 3.

In the description of the microscopic structure of the plant cell walls, we will distinguish between side walls (parts of the cell walls parallel to the $x_3$-axis) and the upper and lower parts of the cell walls which are orthogonal to the $x_3$-axis. In the side walls, we will consider two microscopic structures. First we assume that in the side walls the microfibrils are distributed periodically and are parallel to the cell walls and orthogonal to the $x_3$-axis. Next, motivated by the reorientation of microfibrils in the side walls of the plant cells, observed experimentally in Anderson et al. (2010), we assume that the microfibrils are rotated through the thickness of the side walls and are parallel to the $x_1$ and $x_2$-axes, respectively, at the inner parts of the cell walls and parallel to the $x_3$-axis near the middle lamella.

The subdomains $\Omega _1=(10.16, 20.12)\times (10.16,20.12)\times (27.9, 30.1)$, $\Omega _2=(0,9.96)\times (10.16,20.12)\times (18.6, 20.8)$, $\Omega _3=(0,9.96)\times (0,9.96)\times (9.3,11.5)$, and $\Omega _4=(10.16, 20.12)\times (0, 9.96)\times (18.6, 20.8)$ contain the upper and lower parts of cell walls; see Figs. 2, 3, or 5 for the cross sections. Each domain $\Omega _j$ is divided into a lower part $\Omega _{j,l}$ and upper part $\Omega _{j,u}$, separated by middle lamella, where $j=1,2,3,4$. The length in the $x_1$ and $x_2$-directions of these eight subdomains of the cell walls are $9.96\,\upmu$m, and the thickness of each subdomain (the length in the $x_3$-direction) is $1.0\,\upmu$m. To analyse the impact of the orientation of the microfibrils in the upper and lower parts of the plant cell walls on the elastic deformation of plant cell walls and tissues, we will consider different microfibril orientations within the upper and lower subdomains.

**Fig. 6:**
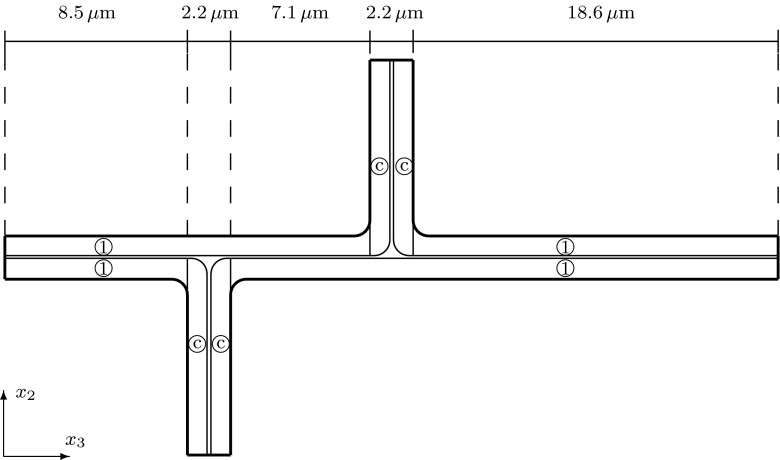
A cross section of $\Omega$ at a constant $x_1$-value in (0, 8.16). All of the rounded corners have a radius of $0.8\;\upmu$m. The regions marked with 1 have cellulose microfibrils parallel to the $x_1$-axis or microfibrils rotated through the thickness of the cell wall and parallel to the $x_1$-axis at the inner part of the cell wall and parallel to the $x_3$-axis near the middle lamella. The regions marked with *c* correspond to the upper and lower parts of the cell walls, and different microfibril orientations will be considered in these regions. The region that is not marked is the middle lamella, which has no microfibrils

### Model Equations and Boundary Conditions

The primary cell walls and the middle lamella are modelled as linearly elastic materials with different elastic properties. Let $\mathbb {E}$ be the elasticity tensor for the cell walls and middle lamella. The value of $\mathbb {E}=\mathbb {E}(x)$ at any given point $x\in \Omega$ depends on where in the plant tissue that point lies, e.g. in the middle lamella or in the cell wall. Moreover, in the different parts of the cell walls, we will consider different orientations of the cellulose microfibrils, which influence the elasticity tensor. This dependence will be specified in detail in the next subsection.

The boundary $\partial \Omega$ of the domain can be split into the union of three sets:

1 $$\begin{aligned} \Gamma _0&=\{x\in \partial \Omega \,| \,x_1=0\,\text {or}\,x_2=0 \,\text {or}\,x_3=0\}, \end{aligned}$$

2 $$\begin{aligned} \Gamma _\text {max}&=\{x\in \partial \Omega \,|\,x_1=x_{1, \mathrm {max}}\,\text {or}\,x_2= x_{2, \mathrm {max}} \,\text {or}\,x_3=x_{3,\mathrm {max}} \}, \end{aligned}$$

3 $$\begin{aligned} \Gamma _I&=\partial \Omega \,\backslash \,(\Gamma _0\cup \Gamma _\text {max}). \end{aligned}$$

The set $\Gamma _I$ is the part of $\partial \Omega$ in contact with the interior of the cells. A pressure boundary condition corresponding to the turgor pressure will be imposed on $\Gamma _{I}$. On $\Gamma _\text {max}$, a tensile traction boundary condition will be specified. Finally, $\Gamma _0$ is the part of the boundary of $\Omega$ that lies on the planes $x_1=0$, $x_2=0$, or $x_3=0$ and we assume the displacement in the normal direction on $\Gamma _0$ to be zero. These boundary conditions reflect the symmetry in the $x_1$- and $x_2$-directions and an assumption that the displacement in the $x_3$-direction is impeded at $x_3=0$, motivated by the fact that the upper part of a plant root system and the lower part of a stem are not moving in the $x_3$-direction and by the experimental set-up where one end of a plant tissue is fixed; see, e.g. Hejnowicz and Sievers (1995). For the symmetric distribution of cells, these boundary conditions also reflect the symmetry in the $x_1$-, $x_2$-, and $x_3$-directions.

Neglecting inertia and external body forces, the elasticity equations with these boundary conditions for the displacement $\mathbf{u}$ are given by

4 $$\begin{aligned} {\left\{ \begin{array}{ll} \text {div}(\mathbb {E}\, \mathbf{e}(\mathbf{u}))=\mathbf 0 &{} \text {in}\,\Omega ,\\ \mathbf{u}\cdot \varvec{\nu }= 0 &{} \text {on}\,\Gamma _0,\\ (\mathbb {E}\, \mathbf{e}(\mathbf{u}))\varvec{\nu }\text { is parallel to }\varvec{\nu }&{} \text {on}\,\Gamma _0,\\ (\mathbb {E}\, \mathbf{e}(\mathbf{u}))\varvec{\nu }= f\varvec{\nu }&{} \text {on}\,\Gamma _\text {max},\\ (\mathbb {E}\, \mathbf{e}(\mathbf{u}))\varvec{\nu }= -p\varvec{\nu }&{} \text {on}\,\Gamma _I, \end{array}\right. } \end{aligned}$$

where $\mathbf{e}(\mathbf{u})=\frac{1}{2}(\nabla \mathbf{u}+\nabla \mathbf{u}^T)$ is the symmetric part of the gradient of the displacement and $\varvec{\nu }$ is the exterior unit normal to $\partial \Omega$. A unique solution of (4) exists in $H^1(\Omega ,\mathbb {R}^3)$, see e.g. Oleinik et al. (1992), provided that $f \in L^2(\Gamma _\text {max})$, $p\in L^2(\Gamma _\text {I})$, and $\mathbb {E}$ satisfies the following conditions:

1. $|\mathbb {E}|$ is bounded in $L^\infty (\Omega )$.
2. There is a strictly positive $\alpha$ such that $\alpha |\mathbf{A}|^2\le \mathbf{A}\cdot \mathbb {E}(x)\mathbf{A}$ for all symmetric $\mathbf{A}\in \mathbb {R}^{3\times 3}$ and $x\in \Omega$.
3. $\mathbb {E}$ possesses major and minor symmetries, i.e. $\mathbb {E}_{ijkl}=\mathbb {E}_{jikl}=\mathbb {E}_{klij}=\mathbb {E}_{ijlk}$.

### The Elasticity Tensor

Next, we specify the elasticity tensor $\mathbb {E}$ on the domain $\Omega$. To do so, we must specify the elasticity tensor for the middle lamella and the cell walls for different microfibril configurations. The macroscopic elastic properties of the cell wall are derived from the microscopic description of the elastic properties of the cell wall matrix and microfibrils using techniques of periodic homogenization. This requires the specification of the elastic properties of the cell wall matrix and the cellulose microfibrils.

The cell wall matrix is isotropic (Zsivanovits et al. 2004), and so the elasticity tensor of the matrix $\mathbb {E}_M$ is of the form

$$\begin{aligned} \mathbb {E}_M\mathbf{A}= 2\mu _M\mathbf{A}+ \lambda _M(\text {tr}\,\mathbf{A})\mathbf 1 , \end{aligned}$$

where the Lamé moduli $\mu _M$ and $\lambda _M$ are related to the Young’s modulus $E_M$ and Poisson’s ratio $\nu _M$ through

$$\begin{aligned} E_M=\frac{\mu _M(2\mu _M+3\lambda _M)}{\mu _M+\lambda _M}\quad \text {and}\quad \nu _M=\frac{\lambda _M}{2(\mu _M+\lambda _M)}. \end{aligned}$$

We take $\nu _M=0.3$, which is common for biological materials, see Baskin and Jensen (2013), Hejnowicz and Sievers (1995), Huang et al. (2012), and Niklas (1992) for more information about the Poisson’s ratio for plant cell walls, and $E_M=5$ MPa. This value is lower than the Young’s modulus measured for highly de-methylesterified pectin gels considered in Zsivanovits et al. (2004) since the pectin within the cell wall matrix is not fully de-esterified.

The cellulose microfibrils are not isotropic (Diddens et al. 2008), so we assume that they are transversely isotropic and, hence, the elasticity tensor $\mathbb {E}_F$ for the microfibrils is determined by specifying five parameters: the Young’s modulus $E_F$ associated with the directions lying perpendicular to the microfibril, the Poisson’s ratio $\nu _{F1}$ characterizing the transverse reduction of the plane perpendicular to the microfibril for stress lying in this plane, the ratio $n_F$ between $E_F$ and the Young’s modulus associated with the direction of the axis of the microfibril, the Poisson’s ratio $\nu _{F2}$ governing the reduction in the plane perpendicular to the microfibril for stress in the direction of the microfibril, and the shear modulus $Z_F$ for planes parallel to the microfibril. A transversely isotropic elasticity tensor expressed in Voigt notation is of the form

$$\begin{aligned} \left( \begin{matrix} \alpha _2+\alpha _5 &{}\quad \alpha _2-\alpha _5 &{}\quad \alpha _3 &{}\quad 0 &{}\quad 0 &{}\quad 0\\ \alpha _2-\alpha _5 &{}\quad \alpha _2+\alpha _5 &{}\quad \alpha _3 &{}\quad 0 &{}\quad 0 &{}\quad 0\\ \alpha _3 &{} \quad \alpha _3 &{}\quad \alpha _1 &{}\quad 0 &{}\quad 0 &{} \quad 0\\ 0 &{} \quad 0 &{}\quad 0 &{} \quad \alpha _4 &{}\quad 0 &{}\quad 0\\ 0 &{}\quad 0 &{}\quad 0 &{}\quad 0 &{}\quad \alpha _4 &{}\quad 0\\ 0 &{}\quad 0 &{}\quad 0 &{}\quad 0 &{}\quad 0 &{}\quad \alpha _5 \end{matrix} \right) , \end{aligned}$$

where $\alpha _i$, for $i=1,2,3,4,5$, are related to the five parameters described above through

$$\begin{aligned} \alpha _1&=\frac{E_F(1-\nu _{F1})}{n_F(1-\nu _{F1})-2\nu _{F2}^2},\qquad \quad \alpha _2=\frac{E_Fn_F}{2n_F(1-\nu _{F1})-4\nu _{F2}^2},\\ \alpha _3&=\frac{E_F\nu _{F2}}{n_F(1-\nu _{F1})-2\nu _{F2}^2},\quad \alpha _4=Z_F,\quad \alpha _5=\frac{E_F}{2(1+\nu _{F1})}. \end{aligned}$$

We assign these parameters the values

$$\begin{aligned} E_F = 15{,}000\, \text {MPa},\,\nu _{F1}= 0.3,\,n_F = 0.068,\,\nu _{F2}=0.06,\,Z_F = 85{,}000 \, \text {MPa}, \end{aligned}$$

which are chosen based on experimental results (Diddens et al. 2008) and to ensure that the elasticity tensor for the microfibrils is positive definite (Nakamura et al. 2004; Padovani 2002).

We assume that the middle lamella is isotropic, with elasticity tensor $\mathbb {E}_{ML}$, and has a Young’s modulus of 15 MPa and Poisson’s ratio of 0.3. It is known from experiments that the density of calcium–pectin cross-links strongly influences the elastic properties of the cell wall matrix and middle lamella (Wolf et al. 2012). Thus, since in the middle lamella almost all pectin is de-esterified and the density of the pectin–calcium cross-links is higher than in the cell wall matrix, where usually only $70\,\%$ of the pectin is de-esterified, we assume that the Young’s modulus for the middle lamella is three times larger than the Young’s modulus for the cell wall matrix.

We first consider that the cellulose microfibrils are arranged periodically within the cell wall matrix (Thomas et al. 2013) and so standard techniques in homogenization theory, see e.g. Oleinik et al. (1992), yield a macroscopic elasticity tensor for a plant cell wall from the microscopic description of the mechanical properties of a cell wall on the level of a single microfibril. In addition to the elastic properties of the microfibrils and cell wall matrix, the macroscopic elasticity tensor depends on the orientation of the cellulose microfibrils. The components of this tensor are determined by solving problems defined on a Representative Volume Element (RVE), in the homogenization literature called the “unit cell” problem, which have the form of the equations of linear elasticity and reflect the arrangement of the microfibrils in different parts of the cell walls. Notice that the multiscale analysis of the microscopic model is preformed for the nondimensionalized model equations and the dimensional quantities are then recovered in the macroscopic equations, while the problem defined on the RVE is dimensionless.

**Fig. 7:**
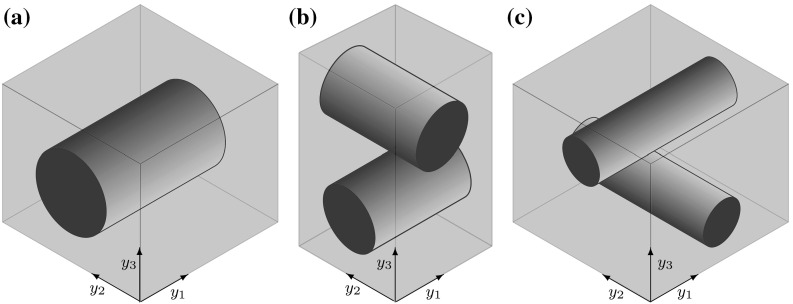
A depiction of the Representative Volume Element (RVE) *Y* with three configurations of microfibrils. **a** A picture of the RVE with one microfibril occupying the set specified in (5). **b** A picture of the RVE with two microfibrils occupying the set specified in (6). **c** A picture of the RVE with two microfibrils occupying the set specified in (7), reflecting a lower density in the distribution of microfibrils in the $y_1$- and $y_2$-directions than in the $y_3$-direction

The microscopic structure in a plant cell wall is determined by the radius and orientation of microfibrils and by the distance between the microfibrils. In the context of homogenization theory, the microstructure of the cell wall is characterized by the configuration of microfibrils in the corresponding RVE. Three types of configurations of microfibrils are considered here:

- there is only one microfibril in the RVE $Y=(0,1)^3$ occupying the set
  5 $$\begin{aligned} Y_F=\{ y \in Y \,|\,(y_2-0.5)^2+(y_3-0.5)^2 < 0.25^2\}, \end{aligned}$$
- there are two perpendicular microfibrils in the RVE $Y=(0, 0.5)^2\times (0,1)$ occupying the set
  6 $$\begin{aligned} Y_{F}= & {} \{y\in Y \,|\,(y_2-0.25)^2+(y_3-0.75)^2<0.125^2\,\text {or}\nonumber \\&(y_1-0.25)^2+(y_3-0.25)^2<0.125^2\}, \end{aligned}$$
- the RVE $Y=(0,1)^3$ with two perpendicular microfibrils occupying the domain
  7 $$\begin{aligned} Y_{F}= & {} \{y\in Y \,|\,(y_2-0.5)^2+(y_3-0.75)^2<0.125^2\,\text {or}\nonumber \\&(y_1-0.5)^2+(y_3-0.25)^2<0.125^2\}, \end{aligned}$$

see Fig. 7. Cases (b) and (c) are similar, except in case (c) the density of the microfibrils in the $y_3$-direction is higher than in the $y_1$- and $y_2$-directions.

We have $\overline{Y} = \overline{Y}_M\cup \overline{Y}_F$, where $Y_M$ and $Y_F$ are disjoint and $Y_M$ represents the part of *Y* occupied by the cell wall matrix. Notice that for the simplicity of presentation we use the same notations for domains *Y*, $Y_M$, and $Y_F$, defining different RVEs and different microfibrils configurations.

The elasticity tensor $\mathbb {E}_Y$ in *Y* is given by

$$\begin{aligned} \mathbb {E}_Y(y)= {\left\{ \begin{array}{ll} \mathbb {E}_M &{} \text {if } y\in Y_M,\\ \mathbb {E}_F &{} \text {if } y\in Y_F, \end{array}\right. } \end{aligned}$$

and can be extended *Y*-periodically to all of $\mathbb {R}^3$. Consider a subdomain *U* of $\Omega$ in which the cellulose microfibrils are arranged periodically with the distribution and orientation specified by the RVE *Y* and $Y_F$ defined in (5), (6), or (7). Let $\varepsilon$ be a small parameter associated with the ratio between the distance between the cellulose microfibrils and the size of *U*. The microfibrils of a plant cell wall are about 3 nm in diameter and are separated by a distance of about 6 nm, see e.g. Colvin (1963), Jennedy et al. (2007), and Thomas et al. (2013), whereas the thickness of a plant cell wall is of the order of a few micrometres. To obtain the elasticity tensor for the part of the cell wall *U* with a periodic microstructure on the length scale of $\varepsilon$ defined by the structure of $\varepsilon Y$, the periodic extension of $\mathbb {E}_Y$ must be scaled appropriately. Namely, the elasticity tensor in *U* is given by

$$\begin{aligned} \mathbb {E}^\varepsilon (x)=\mathbb {E}_Y\left( \frac{x}{\varepsilon }\right) \quad \qquad {\text {for all}}\,\, x\in U. \end{aligned}$$

Then homogenization theory yields a macroscopic elasticity tensor $\mathbb {E}_{\text {hom}}$ that describes a material whose behaviour approximates the behaviour of the cell wall with elasticity tensor $\mathbb {E}^\varepsilon$ when $\varepsilon$ is very small (Oleinik et al. 1992). In our situation $\varepsilon \approx 10^{-3}$. Moreover, $\mathbb {E}_\text {hom}$ is given by8where $\mathbf{w}^{kl}\in H^1(Y,\mathbb {R}^3)$ is the unique solution of

9 $$\begin{aligned} {\left\{ \begin{array}{ll} \text {div}_y\big (\mathbb {E}_Y(\mathbf{e}_y(\mathbf{w}^{kl})+\mathbf{b}^{kl})\big ) = \mathbf 0 \qquad &{} \text {in } Y,\\ \int _Y\mathbf{w}^{kl}\, dy = \mathbf 0 ,\,&{}\mathbf{w}^{kl}\text { is } Y\text {-periodic}, \end{array}\right. } \end{aligned}$$

with $\mathbf{b}^{kl}=\frac{1}{2}(\mathbf{b}^k\otimes \mathbf{b}^l+\mathbf{b}^l\otimes \mathbf{b}^k)$ and $k,l=1,2,3$, where $(\mathbf{b}^1,\mathbf{b}^2,\mathbf{b}^3)$ is the standard basis in $\mathbb {R}^3$.

When $Y_F$ is given by (6) and (7), the elasticity tensor given in (8) will be denoted by $\mathbb {E}^{12}_\text {hom,1}$ and $\mathbb {E}^{12}_\text {hom,2}$, respectively, as there are microfibrils in the $x_1$- and $x_2$-directions, while when $Y_F$ is given by (5) the elasticity tensor defined in (8) will be denoted by $\mathbb {E}^{1}_\text {hom}$ since the microfibrils are pointing in the $x_1$-direction.

Moreover, when $Y_F$ is given by (5), then the microscopic elasticity tensor $\mathbb {E}^\varepsilon$ depends only on the two variables $x_2$ and $x_3$. Hence for this configuration of the microstructure, the elasticity tensor $\mathbb {E}_{Y}$ depends only on $y_2$ and $y_3$ and the solutions of the elliptic problems (9) depend only on $\hat{y}=(y_2, y_3)$. Thus, since $\mathbf{w}^{kl}$ are independent of $y_1$, the problems (9) can be reduced to two-dimensional problems (Ptashnyk and Seguin 2016a). To formulate the reduced problems, we consider $\hat{Y}=(0,1)^2$ and

$$\begin{aligned} \hat{Y}_F = \{ (\hat{y}_2,\hat{y}_3)\in \hat{Y} \,|\,(\hat{y}_2-0.5)^2+(\hat{y}_3-0.5)^2 < 0.25^2\}, \end{aligned}$$

so that $Y=(0,1)\times \hat{Y}$ and $Y_F=(0,1)\times \hat{Y}_F$. It can be shown that10with $\hat{\mathbf{w}}^{kl}\in H^1(\hat{Y},\mathbb {R}^3)$ being the unique solution of

11 $$\begin{aligned} {\left\{ \begin{array}{ll} \hat{\text {div}}_{\hat{y}}\big (\mathbb {E}_Y(0,\hat{y})(\hat{\mathbf{e}}_{\hat{y}}(\hat{\mathbf{w}}^{kl})+\mathbf{b}^{kl})\big ) = \mathbf 0 \qquad &{} \text {in } \hat{Y},\\ \int _{\hat{Y}}\hat{\mathbf{w}}^{kl}\, d\hat{y} = \mathbf 0 ,\,&{}\hat{\mathbf{w}}^{kl}\text { is } \hat{Y}\text {-periodic}, \end{array}\right. } \end{aligned}$$

where for a function $\hat{\mathbf{w}}\in H^1(\hat{Y},\mathbb {R}^3)$, the differential operators $\hat{\mathbf{e}}_{\hat{y}}$ and $\hat{\text {div}}_{\hat{y}}$ are defined by

$$\begin{aligned} \hat{\mathbf{e}}_{\hat{y}}(\hat{\mathbf{w}}) = \left( \begin{matrix} 0 &{} \frac{1}{2}\partial _{y_2} \hat{\mathbf{w}}_1 &{} \frac{1}{2}\partial _{y_3} \hat{\mathbf{w}}_1\\ \frac{1}{2}\partial _{y_2} \hat{\mathbf{w}}_1 &{} \partial _{y_2} \hat{\mathbf{w}}_2 &{} \frac{1}{2}(\partial _{y_2} \hat{\mathbf{w}}_3+\partial _{y_3} \hat{\mathbf{w}}_2)\\ \frac{1}{2}\partial _{y_3}\hat{\mathbf{w}}_1 &{} \frac{1}{2}(\partial _{y_2} \hat{\mathbf{w}}_3+\partial _{y_3} \hat{\mathbf{w}}_2) &{} \partial _{y_3} \hat{\mathbf{w}}_3 \end{matrix} \right) \text { and } \hat{\text {div}}_{\hat{y}}\hat{\mathbf{w}} = \partial _{y_2}\hat{\mathbf{w}}_2+\partial _{y_3}\hat{\mathbf{w}}_3, \end{aligned}$$

see e.g. Ptashnyk and Seguin (2016a). Reducing the dimension of the problem defined on the RVE to two allows for the consideration of a higher-resolution mesh when solving the problem (11) numerically.

Besides considering the macroscopic elasticity tensor for the microstructure defined by microfibrils parallel to the $x_1$-axis, we will also consider the macroscopic elasticity tensor for the microstructure generated by microfibrils that are arranged in other directions in the $x_1x_2$-plane. Given $\theta \in [-\pi /2, \pi /2]$, let $\mathbf{R}^\theta$ denote the rotation about the $x_3$-axis through the angle $\theta$, so that

$$\begin{aligned} \mathbf{R}^\theta =\left( \begin{matrix} \cos \theta &{}\quad \sin \theta &{}\quad 0\\ -\sin \theta &{}\quad \cos \theta &{}\quad 0\\ 0&{}\quad 0&{}\quad 1 \end{matrix} \right) . \end{aligned}$$

The macroscopic elasticity tensor $\mathbb {E}^{1,\theta }_\text {hom}$ for a microstructure consisting of microfibrils aligned in the direction $\mathbf{R}^\theta \mathbf{b}^1$ is given by

12 $$\begin{aligned} \mathbb {E}^{1,\theta }_{\text {hom},ijkl} = \mathbf{R}^{\theta }_{ip}\mathbf{R}^{\theta }_{jq}\mathbf{R}^{\theta }_{kr}\mathbf{R}^{\theta }_{ls}\mathbb {E}^1_{\text {hom},pqrs}. \end{aligned}$$

So, for example, the macroscopic elasticity tensor for a microstructure with microfibrils parallel to the $x_2$-axis is given by $\mathbb {E}^{1,\pi /2}_\text {hom}$.

To summarize, the elasticity tensor $\mathbb {E}$ in the domain $\Omega$ is different in different regions within the cell wall. In Figs. 4 and 6, we specify the regions of the cell walls where the microfibrils are parallel to the $x_1$-axis, i.e. $\mathbb {E}=\mathbb {E}^1_\text {hom}$, and the regions of the primary cell wall where the microfibrils are parallel to the $x_2$-axis, i.e. $\mathbb {E}=\mathbb {E}^{1,\pi /2}_\text {hom}$. Within subregion $\Omega _i$, for $i=1,\dots ,4$, corresponding to the upper and lower parts of the cell walls, see Figs. 1, 2, 3 and 5, the elasticity tensor $\mathbb {E}$ will be set equal to $\mathbb {E}^{i,l}_\text {end}$ and $\mathbb {E}^{i,u}_\text {end}$, for the lower and upper parts respectively, where different choices of $\mathbb {E}^{i,l}_\text {end}$ and $\mathbb {E}^{i,u}_\text {end}$ associated with different microfibril configurations will be considered. Within the middle lamella, there are no microfibrils and $\mathbb {E}=\mathbb {E}_{ML}$. To specify the macroscopic elasticity tensor for the side walls consisting of layers of microfibrils rotated through the thickness of the cell wall, we use a formula similar to (12) with the rotation being about the $x_1$ or $x_2$-axes, respectively, and the angle $\theta$ depending on the spatial position in the cell walls, so that $\theta =0$ at the inner side of the cell wall and $\theta =\pi /2$ near the middle lamella.

It follows from the properties of $\mathbb {E}_M$, $\mathbb {E}_F$, and $\mathbb {E}_{ML}$ that the macroscopic elasticity tensor $\mathbb {E}$ for the plant cell wall and middle lamella satisfies the conditions 1–3 mentioned at the end of Sect. 2.2. Hence problem (4) describing the macroscopic elastic properties of the plant cell walls connected by middle lamella is well posed.

## Results of Numerical Simulations

This section presents the results of the numerical simulations of the problems (9) and (11) necessary to calculate the macroscopic elasticity tensors $\mathbb {E}^1_\text {hom}$, $\mathbb {E}^{12}_\text {hom,1}$, and $\mathbb {E}^{12}_\text {hom,2}$ and the simulations of the system (4) for different configurations of cellulose microfibrils in the cell walls. For the numerical simulations of the system (4), we nondimensionalize the model equations by considering 1 spatial unit to be equal to $2\, \upmu$m and 1 unit for stress to be equal to 1 MPa.

The numerical simulations were performed using FEniCS (Logg et al. 2012; Logg and Wells 2010; Ølgaard and Wells 2010). This involved discretizing the domain using a nonuniform mesh and applying the continuous Galerkin method to solve the equations of linear elasticity. The resulting linear system was solved using the iterative Krylov solver, i.e. the general minimal residual method (GMRES), with an algebraic multigrid preconditioner. The convergence and the stopping criteria for the iterative Krylov solver are characterized by the norm of the residual of the *n*th iteration $r_n=Ax_n-b$ for the corresponding linear system $Ax=b$, obtained by applying the Galerkin method to the system of linear elasticity, which must be smaller than the absolute tolerance parameter, chosen to be $10^{-15}$, and the relative tolerance parameter, chosen to be $10^{-6}$, times the initial residual.

### Numerical Simulations for the Problems Defined on the Representative Volume Element (RVE) that Determine the Macroscopic (Effective) Elasticity Tensor

It was observed experimentally that the calcium–pectin chemistry influences the mechanical properties of the cell wall matrix and middle lamella (Wolf et al. 2012). Hence in general, the elastic properties of the cell wall matrix depend on the density of the calcium–pectin cross-links *n* and the microscopic elasticity tensor $\mathbb {E}^\varepsilon$ of the plant cell wall is a function of *n*. It was shown in Ptashnyk and Seguin (2016a) that under the assumption of an isotropic cell wall matrix, the macroscopic elasticity tensor $\mathbb {E}_\text {hom}$ corresponding to any microfibril configuration is an affine function of the Young’s modulus of the cell wall matrix. From experiments (Zsivanovits et al. 2004), it is known that the Young’s modulus $E_M$ of the cell wall matrix is a function of the density of the calcium–pectin cross-links *n* through the formula

13 $$\begin{aligned} E_M=0.775n+8.08, \end{aligned}$$

where $E_M$ has the units of MPa and *n* has the units of $\upmu$M. Thus, knowing the macroscopic elasticity tensor $\mathbb {E}_\text {hom}$ for two different values of $E_M$, we can determine the tensor for any value of $E_M$. Then, using (13), we obtain the macroscopic elasticity tensor for the cell wall for any calcium–pectin cross-links density *n*. This approach enables us to analyse the changes in the mechanical properties of plant cell walls and tissues in response to the dynamics of calcium–pectin chemistry and changes in calcium–pectin cross-link density, which will be the subject of future research.

To obtain the macroscopic elasticity tensor, we first calculate numerically $\mathbb {E}_\text {hom}(E_M)$ for two Young’s moduli $E_M=10$ and $E_M=20$. Then using the fact that $\mathbb {E}_\text {hom}=\mathbb {E}_\text {hom}(E_M)$ is an affine function, we can determine $\mathbb {E}_\text {hom}$ for any value of $E_M$, in particular for $E_M=5$.

To determine $\mathbb {E}^1_\text {hom}$, the RVE $\hat{Y}$ was discretized by a mesh with 18, 645, 460 vertices with a higher density of vertices near the boundary between the cell wall matrix and the microfibrils. Using Voigt notation, the resulting macroscopic (effective) elasticity tensors $\mathbb {E}^1_\text {hom}(E_M)$ for $E_M=10$ and 20 are shown in Table 1, to two decimal places. Using the symmetry of the microstructure, it can be shown analytically that the macroscopic elasticity tensors have tetragonal symmetry (Ptashnyk and Seguin 2016b), meaning that the entries of the matrices $\mathbf{C}^1(10)$ and $\mathbf{C}^1(20)$ that are zero are exact and that some of the coefficients of the matrices $\mathbf{C}^1(10)$ and $\mathbf{C}^1(20)$ are equal. Specifically, for $E_M=10$ or 20, $\mathbf{C}^1(E_M)_{22}$ and $\mathbf{C}^1(E_M)_{33}$ should be equal, $\mathbf{C}^1(E_M)_{12}$ and $\mathbf{C}^1(E_M)_{13}$ should be equal, and $\mathbf{C}^1(E_M)_{55}$ and $\mathbf{C}^1(E_M)_{66}$ should be equal. The largest scale involved in the numerical computations of the macroscopic elasticity tensors is determined by the Young’s modulus of the microfibrils in the direction of the microfibrils and is equal to $2.2 \times 10^5$ MPa. Using this scale, the relative error (the difference divided by $2.2 \times 10^5$) associated with $\mathbf{C}^1(E_M)_{55}$ and $\mathbf{C}^1(E)_{66}$ not being equal is on the order of $10^{-8}$.

**Table 1:** The macroscopic (effective) elasticity tensor $\mathbb {E}^1_\text {hom}$ expressed in Voigt notation to two decimal places when the Young’s modulus of the cell wall matrix is 10 and 20 MPa, respectively

$$\mathbf{C}^1(10)=\left( \begin{matrix} 43333.24 &{} 12.51 &{} 12.51 &{} 0 &{} 0 &{} 0\\ 12.51 &{} 19.27 &{} 7.59 &{} 0 &{} 0 &{} 0 \\ 12.50 &{} 7.59 &{} 19.27 &{} 0 &{} 0 &{} 0 \\ 0 &{} 0 &{} 0 &{} 5.34 &{} 0 &{} 0\\ 0 &{} 0 &{} 0 &{} 0 &{} 9.30 &{} 0 \\ 0 &{} 0 &{} 0 &{} 0 &{} 0 &{} 9.32 \end{matrix}\right) $$
$$\mathbf{C}^{1}(20)= \left( \begin{matrix} 43352.40 &{} 24.07 &{} 24.07 &{} 0 &{} 0 &{} 0 \\ 24.07 &{} 37.75 &{} 14.89 &{} 0 &{} 0 &{} 0 \\ 24.07 &{} 14.89 &{} 37.75 &{} 0 &{} 0 &{} 0 \\ 0 &{} 0 &{} 0 &{} 10.44 &{} 0 &{} 0 \\ 0 &{} 0 &{} 0 &{} 0 &{} 15.04 &{} 0 \\ 0 &{} 0 &{} 0 &{} 0 &{} 0 &{} 15.05 \end{matrix}\right) $$

For the numerical calculations of the effective elasticity tensors for the microscopic structures given by the RVE *Y* and the domain occupied by microfibrils $Y_{F}$ defined in (6) and (7), respectively, we discretize *Y* by a mesh with 9, 177, 795 vertices in the case of (6) and 11, 750, 289 vertices in the case of (7), with a higher density of vertices near the boundary between the cell wall matrix and the microfibrils. The calculated macroscopic elasticity tensors $\mathbb {E}^{12}_\text {hom,1}(E_M)$ for microfibrils configuration given by (6) and $\mathbb {E}^{12}_\text {hom,2}(E_M)$ for microfibrils configuration as in (7), where $E_M=10$ or $E_M=20$, are shown in Tables 2 and 3 using Voigt notation. Similar to the results in the previous paragraph, the macroscopic elasticity tensors should have tetragonal symmetry. The largest relative error associated with the components expected to be equal is on the order of $10^{-5}$.

**Table 2:** The macroscopic (effective) elasticity tensor $\mathbb {E}^{12}_\text {hom,1}$ for a part of the cell wall with the microscopic structure defined by the REV in which $Y_F$ is specified by (6), expressed in Voigt notation to two decimal places when the Young’s modulus of the cell wall matrix is 10 and 20 MPa, respectively

$$\mathbf{C}^{12}_1(10)= \left( \begin{matrix} 21715.44 &{} 68.14 &{} 45.82 &{} 0 &{} 0 &{} 0\\ 68.14 &{} 21715.43 &{} 46.73 &{} 0 &{} 0 &{} 0\\ 45.82 &{} 46.73 &{} 64.85 &{} 0 &{} 0 &{} 0\\ 0 &{} 0 &{} 0 &{} 122.43 &{} 0 &{} 0\\ 0 &{} 0.02 &{} 0 &{} 0 &{} 117.82 &{} 0\\ 0 &{} 0 &{} 0 &{} 0 &{} 0 &{} 220.50 \\ \end{matrix}\right) $$
$$\mathbf{C}^{12}_1(20)=\left( \begin{matrix} 21733.92 &{} 79.87 &{} 55.22 &{} 0 &{} 0 &{} 0\\ 79.87 &{} 21733.92 &{} 56.13 &{} 0 &{} 0 &{} 0\\ 55.22 &{} 56.13 &{} 83.12 &{} 0 &{} 0 &{} 0\\ 0 &{} 0 &{} 0 &{}127.83 &{} 0 &{} 0\\ 0 &{} 0 &{} 0 &{} 0 &{} 123.15 &{} 0\\ 0 &{} 0 &{} 0 &{} 0 &{} 0 &{} 226.23\\ \end{matrix}\right) $$

**Table 3:** The macroscopic (effective) elasticity tensor $\mathbb {E}^{12}_\text {hom,2}$ for a part of the cell wall with the microscopic structure defined by the REV in which $Y_F$ is specified by (7), expressed in Voigt notation to two decimal places when the Young’s modulus of the cell wall matrix is 10 and 20 MPa, respectively

$$\mathbf{C}^{12}_2(10)=\left( \begin{matrix} 10927.86 &{} 99.60 &{} 67.85 &{} 0 &{} 0 &{} 0\\ 99.60 &{} 10927.69 &{} 66.46 &{} 0 &{} 0 &{} 0\\ 67.85 &{} 66.46 &{} 91.00 &{} 0 &{} 0 &{} 0\\ 0 &{} 0 &{} 0 &{} 186.83 &{} 0 &{} 0\\ 0 &{} 0 &{} 0 &{} 0 &{} 193.97 &{} 0\\ 0 &{} 0 &{} 0 &{} 0 &{} 0 &{} 352.58 \\ \end{matrix}\right) $$
$$\mathbf{C}^{12}_2(20)= \left( \begin{matrix} 10943.35 &{} 107.84 &{} 75.25 &{} 0 &{} 0 &{} 0\\ 107.84 &{} 10943.18 &{} 73.87 &{} 0 &{} 0 &{} 0\\ 75.25 &{} 73.87 &{} 106.55 &{} 0 &{} 0 &{} 0\\ 0 &{} 0 &{} 0 &{}191.43 &{} 0 &{} 0\\ 0 &{} 0 &{} 0 &{} 0 &{} 198.56 &{} 0\\ 0 &{} 0 &{} 0 &{} 0 &{} 0 &{} 357.20\\ \end{matrix}\right) $$

The results of this section allow us to compute the elasticity tensor for any Young’s modulus of the cell wall matrix; however, in the following analysis, we only consider the case where $E_M=5$ MPa.

### Numerical Simulations of Problem (4) for Different Boundary Conditions and Microfibril Orientations in the Upper and Lower Parts of Cell Walls and in the Side Walls

Using the numerical results for the effective elasticity tensor for different microfibril orientations, in this section we consider different microfibril orientations in the eight subregions corresponding to the upper and lower parts of the cell walls and different specifications of the turgor pressure *p* and tensile force *f* in problem (4). We also consider two scenarios for the microfibril orientation in the side walls: (a) the microfibrils are parallel to the cell walls and orthogonal to the $x_3$-axis and (b) the layers of the microfibrils are rotated through the cell wall thickness.

We consider two different choices for *p* in the boundary conditions in (4). For the pressure inside the cells, we set $p=p_{\circ ,j}$, $j=1,2$, with $p_{\circ ,1}=0.209$ MPa or $p_{\circ ,2}=0.3$ MPa, which are common values for the turgor pressure in plant cells (Benkert et al. 1997; Dyson et al. 2014). For the tensile traction condition in (4), following the experimental results in Hejnowicz and Sievers (1995), we consider the force $f_{ex}=0.049$ N acting on 1 mm of circumference plant tissue surface. This corresponds to $f= 0.614= 2.938 p_{\circ ,1}$ MPa and $f= 2.047p_{\circ ,2}$ MPa, respectively. A similar value for a force acting on the ends of a part of a cell wall was used in Huang et al. (2012) by assuming that $f=(r/2\delta ) p_{\circ ,1}$, where *r* denotes the inner radius of the cell and $\delta$ the thickness of the cell wall. For our geometry, this formula gives $f=4.48 p_{\circ ,1}$.

For the boundary conditions, we considered the following four cases:

(BC1): Base case: $p=p_{\circ ,1}=0.209$ MPa and $f=2.938 p_{\circ ,1}$ MPa.
(BC1$$^\prime $$)
Different turgor pressure: $p=p_{\circ ,2}=0.3$ MPa and $f= 2.047p_{\circ ,2}$ MPa.
(BC2): No tensile tractions: $p=p_{\circ , 1}$ and $f=0$.
(BC3): Different turgor pressures in neighbouring cells and no tensile tractions: $p_1=p_4=p_5=p_8=p_{\circ , 1}$ and $p_2=p_3=p_6=p_7=1.3p_{\circ ,1}$, where $p_i$, for $i=1,\dots ,8$, is the pressure in cell *i*, and $f=0$.

**Fig. 8:**
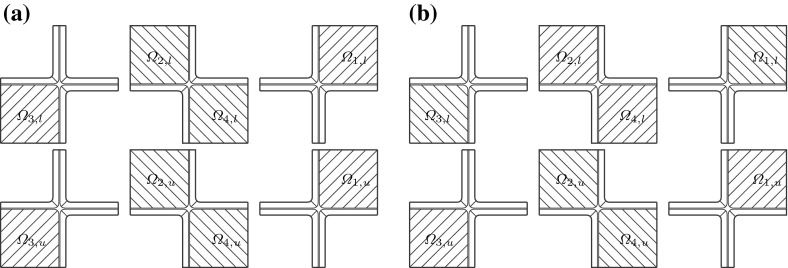
**a** A depiction of the orientation of the cellulose microfibrils in (C1). **b** A depiction of the orientation of the cellulose microfibrils in (C2)

For each of these boundary conditions, we consider five different configurations of the microfibrils in the eight subregions corresponding to the lower and upper parts of the cell walls; see Fig. 8.

(C1): In subregions $\Omega _{1,l}$, $\Omega _{1,u}$, $\Omega _{3,l}$ and $\Omega _{3,u}$ the microfibrils are parallel to $\mathbf{R}^{\pi /4}\mathbf{b}^1$ and in subregions $\Omega _{2,l}$, $\Omega _{2,u}$, $\Omega _{4,l}$ and $\Omega _{4,u}$ the microfibrils are parallel to $\mathbf{R}^{-\pi /4}\mathbf{b}^1$. Thus, $\mathbb {E}^{i,l}_\text {end}= \mathbb {E}^{i,u}_\text {end}=\mathbb {E}^{1,\pi /4}_\text {hom}$ for $i=1, 3$, and $\mathbb {E}^{i,l}_\text {end}= \mathbb {E}^{i,u}_\text {end}=\mathbb {E}^{1,-\pi /4}_\text {hom}$ for $i=2, 4$, see Fig. 8a.
(C2): In subregions $\Omega _{2,l}$, $\Omega _{4,l}$, $\Omega _{1,u}$ and $\Omega _{3,u}$ the microfibrils are parallel to $\mathbf{R}^{\pi /4}\mathbf{b}^1$ and in subregions $\Omega _{1,l}$, $\Omega _{3,l}$, $\Omega _{2,u}$ and $\Omega _{4,u}$ the microfibrils are parallel to $\mathbf{R}^{-\pi /4}\mathbf{b}^1$. Thus $\mathbb {E}^{2,l}_\text {end}=\mathbb {E}^{4,l}_\text {end}=\mathbb {E}^{1,u}_\text {end}=\mathbb {E}^{3,u}_\text {end}=\mathbb {E}^{1,\pi /4}_\text {hom}$ and $\mathbb {E}^{1,l}_\text {end}=\mathbb {E}^{3,l}_\text {end}=\mathbb {E}^{2,u}_\text {end} =\mathbb {E}^{4,u}_\text {end}=\mathbb {E}^{1,-\pi /4}_\text {hom}$, see Fig. 8b.
(C3): In all of the eight subregions, the orientations of the microfibrils on the microscale are generated by the RVE depicted in Fig. 7b. Thus, $\mathbb {E}^{i,l}_\text {end}=\mathbb {E}^{i,u}_\text {end}=\mathbb {E}^{12}_\text {hom,1}$ for $i=1,\dots ,4$.
(C3$$^\prime $$)
In all of the eight subregions, the orientations of the microfibrils on the microscale are generated by the RVE depicted in Fig. 7c. Thus, $\mathbb {E}^{i,l}_\text {end}=\mathbb {E}^{i,u}_\text {end}=\mathbb {E}^{12}_\text {hom,2}$ for $i=1,\dots ,4$.
(C4): There are no microfibrils in the upper and lower parts of the cell walls. Instead, the upper and lower parts of the cell walls consist of middle lamella and, hence, $\mathbb {E}^{i,l}_\text {end}=\mathbb {E}^{i,u}_\text {end}=\mathbb {E}_{ML}$ for $i=1,\dots ,4$.

As a base case for the geometry, we consider the domain $\Omega$ depicted in Fig. 2. For the numerical simulations, we discretize the domain $\Omega$ with a mesh comprising 12, 143, 330 vertices with a higher density of vertices within the subdomains corresponding to the lower and upper parts of the cell walls and near the round edges of the cell walls.

**Fig. 9:**
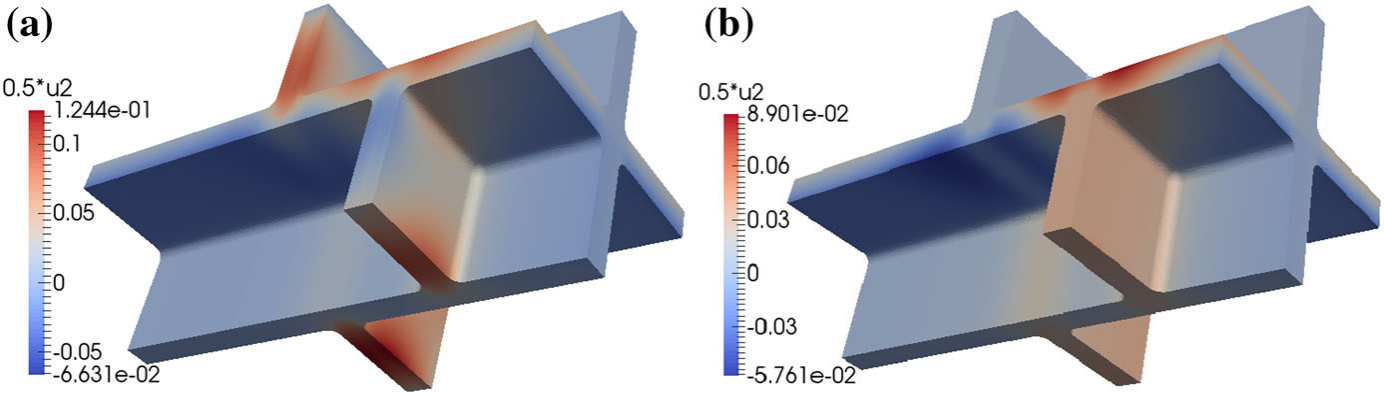
A depiction of the displacements in the $x_2$-direction for (BC1) with two different microfibril configurations: **a** configuration (C1) and **b** configuration (C3). Here the base case for the geometry, as depicted in Fig. 2, is used and the microfibrils in the side walls are orthogonal to the $x_3$-axis

**Fig. 10:**
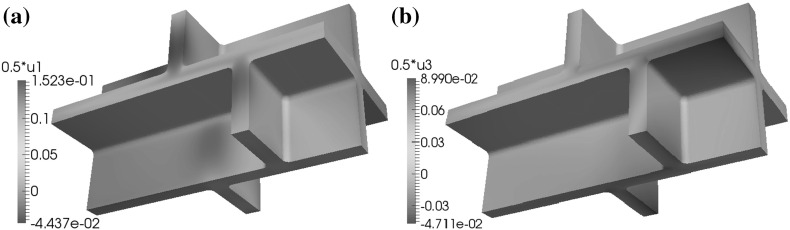
A depiction of the displacement **a** in the $x_1$-direction and **b** in the $x_3$-direction, for boundary conditions (BC1) and configuration (C1), in the case where the geometry is as shown in Fig. 2 (base case) and the microfibrils are rotated through the thickness of the side walls

The results of the numerical simulations of the system (4) with the boundary conditions (BC1)–(BC3) for the base case of the geometry for the configurations (C1)–(C4) in the upper and lower parts of the cell walls and the microfibrils in the side walls oriented orthogonal to the $x_3$-axis are shown in Tables 4, 6, 7, and 8 and Figs. 9 and 12. For the boundary condition (BC1) and the configurations (C1)–(C4), we also consider the microscopic structure in the side walls defined by the layers of microfibrils rotated through the wall thickness; see Table 5 and Fig. 10.

**Table 4:** The maximum and minimum values of the diagonal components of the strain tensor, divergence of the displacement, and the maximal displacement in the positive and negative $x_1$-, $x_2$-, and $x_3$-directions, to four significant figures, for boundary condition (BC1), configurations (C1)–(C4), for the base case of the domain $\Omega$, as in Fig. 2, and the microfibrils (MF) in the side walls are orthogonal to the $x_3$-axis

(BC1), parallel MF	$\mathbf{e}_{11}$		$\mathbf{e}_{22}$		$\mathbf{e}_{33}$		$\mathrm{div } \mathbf{u}$	
$f=2.938p_{\circ ,1}, p_{\circ ,1}=0.209$	Negative	Positive	Negative	Positive	***Negative***	***Positive***	Negative	Positive
(C1)	$$-0.1977$$	0.1902	$$-0.1736$$	0.2051	−***0.0625***	***0.2060***	$$-0.1816 $$	0.2473
(C2)	$-0.1899$	0.2284	$-0.1543$	0.1990	−***0.1787***	***0.2281***	$-0.1317$	0.3050
(C3)	$-0.1500$	0.2797	$-0.2486$	0.2087	−***0.0513***	***0.2122***	$-0.1742$	0.3057
(C3$^\prime$)	$-0.1433$	0.2812	$-0.2602$	0.2095	−***0.0515***	***0.2110***	$-0.1795$	0.3099
(C4)	$-0.2197$	0.2308	$-0.1918$	0.1794	−***0.0471***	***0.2750***	$-0.2285$	0.2870

(BC1), parallel MF	$x_1$-direction		$x_2$-direction		$x_3$-direction			
$f=2.938p_{\circ ,1}, p_{\circ ,1}=0.209$	Negative	*Positive*	Negative	*Positive*	Negative	**Positive**		
(C1)	$-0.1329$	*0.2500*	$-0.1326$	*0.2488*	$-2.752\times 10^{-10}$	**2.560**		
(C2)	$-0.1263$	*0.2764*	$-0.1260$	*0.2686*	$-3.888\times 10^{-9}$	**2.542**		
(C3)	$-0.1153$	*0.1715*	$-0.1152$	*0.1780*	$-1.425\times 10^{-8}$	**2.456**		
(C3$^\prime$)	$-0.1162$	*0.1709*	$-0.1161$	*0.1754*	$-1.681\times 10^{-8}$	**2.448**		
(C4)	$-0.1326$	*0.6602*	$-0.1323$	*0.6592*	$-1.039\times 10^{-8}$	**2.526**		

**Table 5:** The maximum and minimum values of the diagonal components of the strain tensor, divergence of the displacement, and the maximal displacement in the positive and negative $x_1$, $x_2$, and $x_3$-directions, to four significant figures, for boundary condition (BC1), configurations (C1)–(C4), for the base case of the domain $\Omega$, as in Fig. 2, and the microstructure in the side walls is given by the rotated layers of microfibrils (MF)

(BC1), rotated MF	$\mathbf{e}_{11}$		$\mathbf{e}_{22}$		$\mathbf{e}_{33}$		$\mathrm{div } \mathbf{u}$	
$f=2.938p_{\circ ,1}, p_{\circ ,1}=0.209$	Negative	Positive	Negative	Positive	***Negative***	***Positive***	Negative	Positive
(C1)	$-0.1804$	0.2290	$-0.2261$	0.3571	−***0.7740***	***0.7468***	$-0.4285$	0.7588
(C2)	$-0.1642$	0.2102	$-0.1968$	0.3765	−***0.8081***	***0.6684***	$-0.4385$	0.6808
(C3)	$-0.3234$	0.3068	$-0.2971$	0.3451	−***0.7470***	***0.7112***	$-0.4182$	0.7255
(C4)	$-0.1949$	0.2269	$-0.2198$	0.3454	−***0.7354***	***0.6655***	$-0.3807$	0.6784

(BC1), rotated MF	$x_1$-direction		$x_2$-direction		$x_3$-direction			
$f=2.938p_{\circ ,1}, p_{\circ ,1}=0.209$	Negative	*Positive*	Negative	*Positive*	Negative	**Positive**		
(C1)	$-0.0887$	*0.3046*	$-0.0539$	*0.2818*	$-0.0942$	**0.1798**		
(C2)	$-0.0663$	*0.3372*	$-0.0560$	*0.3310*	$-0.5814$	**0.1819**		
(C3)	$-0.0326$	*0.1686*	$-0.0308$	*0.1509*	$-0.0340$	**0.1492**		
(C4)	$-0.0734$	*0.7368*	$-0.0434$	*0.7014*	$-0.0692$	**0.1959**		

**Table 6:** The maximum and minimum values of the diagonal components of the strain tensor, divergence of the displacement, and the maximal displacement in the positive and negative $x_1$-, $x_2$-, and $x_3$-directions, to four significant figures, for boundary condition (BC2), for configurations (C1)–(C4), for the base case of the geometry $\Omega$, as in Fig. 2, and the microfibrils in the side walls are orthogonal to the $x_3$-axis

(BC2)	$\mathbf{e}_{11}$		$\mathbf{e}_{22}$		$\mathbf{e}_{33}$		$\mathrm{div } { \mathbf u}$	
	Negative	Positive	Negative	Positive	Negative	Positive	Negative	Positive
(C1)	$-0.0620$	0.0300	$-0.0663$	0.0468	$-0.0292$	0.0648	$-0.0752$	0.0298
(C2)	$-0.0452$	0.0287	$-0.0437$	0.0290	$-0.0719$	0.0630	$-0.0519$	0.0279
(C3)	$-0.0433$	0.0251	$-0.0436$	0.0255	$-0.0159$	0.0469	$-0.0462$	0.0304
(C3$^\prime$)	$-0.0425$	0.0238	$-0.0428$	0.0257	$-0.0169$	0.0460	$-0.0461$	0.0284
(C4)	$-0.0516$	0.0249	$-0.0493$	0.0262	$-0.0208$	0.0660	$-0.0630$	0.0313

(BC2)	$x_1$-direction		$x_2$-direction		$x_3$-direction			
	Negative	Positive	Negative	Positive	Negative	**Positive**		
(C1)	$-0.0796$	0.0893	$-0.0797$	0.0915	$-5.880\times 10^{-11}$	**0.2690**		
(C2)	$-0.0669$	0.0670	$-0.0664$	0.0680	$-5.020\times 10^{-11}$	**0.2644**		
(C3)	$-0.0435$	0.0507	$-0.0436$	0.0522	$-6.814\times 10^{-10}$	**0.2648**		
(C3$^\prime$)	$-0.0432$	0.0495	$-0.0433$	0.0509	$-4.456\times 10^{-10}$	**0.2658**		
(C4)	$-0.0697$	0.1096	$-0.0700$	0.1104	$-4.948\times 10^{-10}$	**0.2744**		

**Table 7:** The maximum and minimum values for the diagonal components of the strain tensor, divergence of the displacement, and the maximal displacement in the positive and negative $x_1$-, $x_2$-, and $x_3$-directions, to four significant figures, for boundary condition (BC3), for configurations (C1)–(C4), for the base case of the geometry $\Omega$, as in Fig. 2, and the microfibrils in the side walls are orthogonal to the $x_3$-axis

(BC3)	$\mathbf{e}_{11}$		$\mathbf{e}_{22}$		$\mathbf{e}_{33}$		$\mathrm{div }{} \mathbf{u}$	
	Negative	Positive	Negative	Positive	Negative	Positive	Negative	Positive
(C1)	$-0.2835$	0.2090	$-0.2645$	0.1373	$-0.0479$	0.1218	$-0.3255$	0.1369
(C2)	$-0.2821$	0.2015	$-0.1615$	0.1343	$-0.0720$	0.1232	$-0.1541$	0.1315
(C3)	$-0.2821$	0.1825	$-0.1596$	0.1289	$-0.0539$	0.1518	$-0.1556$	0.1482
(C4)	$-0.2840$	0.1440	$-0.1553$	0.1303	$-0.0441$	0.1231	$-0.1550$	0.1283

(BC3)	$x_1$-direction		$x_2$-direction		$x_3$-direction			
	Negative	Positive	Negative	*Positive*	Negative	**Positive**		
(C1)	$-0.0356$	1.1518	$-0.5758$	*0.1572*	$-6.442\times 10^{-11}$	**0.3208**		
(C2)	$-0.0390$	1.1484	$-0.5602$	*0.1575*	$\underline{-0.0259}$	**0.3242**		
(C3)	$-0.0352$	1.1264	$-0.5116$	*0.1038*	$-1.434\times 10^{-9}$	**0.3274**		
(C4)	$-0.0355$	1.1590	$-0.5214$	*0.2244*	$-1.920\times 10^{-9}$	**0.3194**		

**Table 8:** The maximal positive and negative values of the diagonal components of the strain tensor, divergence of the displacement, and the maximal displacement in the positive and negative $x_1$-, $x_2$-, and $x_3$-directions, to four significant figures, for boundary condition (BC1$^\prime$), configurations (C1)–(C4), the domain $\Omega$ as in Fig. 2, and the microfibrils in the side walls are orthogonal to the $x_3$-axis

(BC1$^\prime$)	$\mathbf{e}_{11}$		$\mathbf{e}_{22}$		$\mathbf{e}_{33}$		$\mathrm{div } { \mathbf u}$	
$f=2.047p_{\circ ,2}$, $p_{\circ ,2}=0.3$	Negative	Positive	Negative	Positive	Negative	Positive	Negative	Positive
(C1)	$$-0.2152$$	0.3343	$$-0.2132$$	0.2362	$$-0.0740$$	0.2425	$$-0.1988 $$	0.3304
(C2)	$-0.2040$	0.2338	$-0.1691$	0.2066	$-0.1954$	0.2473	$-0.1477$	0.3166
(C3)	$-0.1739$	0.2942	$-0.2733$	0.2183	$-0.0580$	0.2260	$-0.1955$	0.3186
(C4)	$-0.2401$	0.2341	$-0.2103$	0.1857	$-0.0524$	0.2967	$-0.2482$	0.2938

(BC1$^\prime$)	$x_1$-direction		$x_2$-direction		$x_3$-direction			
$f=2.047p_{\circ ,2}$, $p_{\circ ,2}=0.3$	Negative	*Positive*	Negative	*Positive*	Negative	Positive		
(C1)	$-0.1651$	*0.2628*	$-0.1647$	*0.2610*	$-1.006\times 10^{-8}$	2.678		
(C2)	$-0.1518$	*0.2904*	$-0.1513$	*0.2828*	$-5.986\times 10^{-9}$	2.658		
(C3)	$-0.1335$	*0.1935*	$-0.1334$	*0.2000*	$-1.018\times 10^{-8}$	2.572		
(C4)	$-0.1610$	*0.7082*	$-0.1606$	*0.7074*	$-1.960\times 10^{-8}$	2.646		

**Fig. 11:**
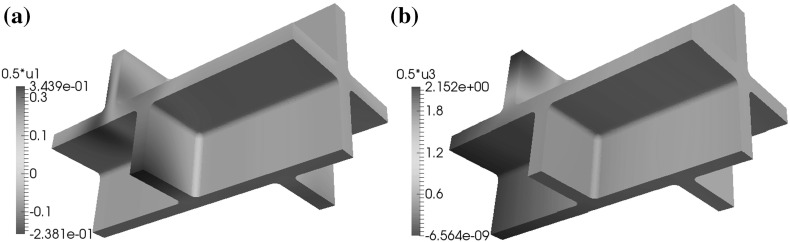
A depiction of the displacements **a** in the $x_1$-direction and **b** in the $x_3$-direction, for the boundary conditions (BC1) and configuration (C4), in the case where the geometry is as shown in Fig. 3 and the microfibrils in the side walls are orthogonal to the $x_3$-axis

We also performed numerical simulations of the model equations (4) for the domain $\Omega$ without a shift in the positions of neighbouring cells, see Fig. 1, and for the domain $\Omega$ where the positions of all pairs of the upper and lower cells are shifted relative to each other; see Fig. 3. The results of the numerical simulations for these two cases are presented in Tables 9 and 10 and Fig. 11.

In the base case for the geometry $\Omega$ and if the microfibrils in the side walls are orthogonal to the $x_3$-axis, for the configurations (C1), (C2), and (C4) and boundary conditions (BC1) and (BC2), the maximal displacements in the $x_1$ and $x_2$-directions occur in the upper and lower parts of the cell walls, while for configuration (C3) and all boundary conditions, the maximal displacements in these directions occur in the side walls near the upper and lower parts of the cell walls, see Fig. 9. For boundary condition (BC3) and configurations (C1), (C2), and (C4), the maximal positive values for the displacement in the $x_1$-direction occur on the side walls, while for the $x_2$-direction the maximal positive values occur on the side walls and the upper and lower parts of the cell walls. For the $x_3$-direction, the maximal displacement occurs on the plane $x_3=x_{3,\mathrm{max}}$, i.e. $x_3=39.4$. For boundary condition (BC3) and configuration (C2), we observe that the nonzero values of the displacement in the negative $x_3$-direction occur in the upper and lower parts of the cell walls.

**Table 9:** The maximal positive and negative values of the diagonal components of the strain tensor, divergence of the displacement, and the maximal values of the displacement in the positive and negative $x_1$-, $x_2$-, and $x_3$-directions, to four significant figures, for the boundary condition (BC1), configurations (C1)–(C4), for the geometry shown in Fig. 3, and the microfibrils in the side walls are orthogonal to the $x_3$-axis

(BC1), 4 shifts	$\mathbf{e}_{11}$		$\mathbf{e}_{22}$		$\mathbf{e}_{33}$		$\mathrm{div } \mathbf{u}$	
$f=2.938p_{\circ ,1}, p_{\circ ,1}=0.209$	Negative	Positive	Negative	Positive	***Negative***	***Positive***	Negative	Positive
(C1)	$-0.3251$	0.5635	$-0.3851$	0.3458	−***0.1541***	***0.3549***	$-0.3574$	0.3809
(C3)	$-0.1639$	0.2154	$-0.2875$	0.2211	−***0.1077***	***0.2309***	$-0.2942$	0.3345
(C4)	$-0.2180$	0.2144	$-0.2525$	0.2785	−***0.1257***	***0.4717***	$-0.2185$	0.3167

(BC1), 4 shifts	$x_1$-direction		$x_2$-direction		$x_3$-direction			
$f=2.938p_{\circ ,1}, p_{\circ ,1}=0.209$	*Negative*	*Positive*	*Negative*	*Positive*	Negative	Positive		
(C1)	−*0.3122*	*0.2670*	−*0.3154*	*0.2676*	$-4.700\times 10^{-9}$	2.752		
(C3)	−*0.1500*	*0.1601*	−*0.1494*	*0.2314*	$-1.838\times 10^{-8}$	2.632		
(C4)	−*0.4762*	*0.6878*	−*0.5080*	*0.6778*	$-1.313\times 10^{-8}$	4.304		

**Table 10:** The maximal negative and positive values of the diagonal components of the strain tensor and the divergence and the maximal displacement in the positive and negative $x_1$-, $x_2$-, and $x_3$-directions, to four significant figures, for the boundary condition (BC1), with $f=2.938p_{\circ ,1}$ and $p_{\circ ,1} =0.209$ MPa, for the microfibril configurations (C1)–(C4) in the lower and upper parts of cell walls, for the eight-cell geometry without a shift in the position of the neighbouring cells, see Fig. 1, and the microfibrils in the side walls are orthogonal to the $x_3$-axis

(BC1), no shift	$\mathbf{e}_{11}$		$\mathbf{e}_{22}$		$\mathbf{e}_{33}$		$\mathrm{div } \mathbf{u}$	
$f=2.938p_{\circ ,1}, p_{\circ ,1}=0.209$	Negative	Positive	Negative	Positive	Negative	Positive	Negative	Positive
(C1)	$-0.1806$	0.2824	$-0.1880$	0.2665	$-0.1718$	0.1751	$-0.2339$	0.3686
(C2)	$-0.1110$	0.2684	$-0.1368$	0.2590	$-0.1980$	0.1613	$-0.1759$	0.2651
(C3)	$-0.0786$	0.1075	$-0.0806$	0.1002	$-0.0488$	0.1438	$-0.0605$	0.1028
(C4)	$-0.1480$	0.1511	$-0.1418$	0.1577	$-0.0736$	0.1898	$-0.2365$	0.2472

(BC1), no shift	$x_1$-direction		$x_2$-direction		$x_3$-direction			
$f=2.938p_{\circ ,1}, p_{\circ ,1}=0.209$	Negative	*Positive*	Negative	*Positive*	Negative	Positive		
(C1)	$-0.0716$	*0.2152*	$-0.0732$	*0.2152*	$-3.184\times 10^{-9}$	2.520		
(C2)	$-0.0653$	*0.2502*	$-0.0646$	*0.2520*	$-2.546\times 10^{-10}$	2.518		
(C3)	$-0.0617$	*0.0595*	$-0.0622$	*0.0590*	$-1.057\times 10^{-9}$	2.480		
(C4)	$-0.0616$	*0.4788*	$-0.0629$	*0.4788*	$-6.596\times 10^{-9}$	2.490		

If the microfibrils in the side walls are rotated through the thickness of the wall, for the (C1) and (C2) configurations and the boundary condition (BC1) the maximal displacements in the positive and negative $x_1$- and $x_2$-directions occur in the upper and lower parts of the cell walls, whereas the maximal displacement in the $x_3$-direction occurs on the side walls and the maximal displacement in the negative $x_3$-direction occurs in the lower parts of the cell walls; see Fig. 10. For configuration (C3) the maximal displacements in the positive and negative $x_1$-, $x_2$-, and $x_3$-directions occur in the side walls. For configuration (C4) the maximal displacements in the positive $x_1$-, $x_2$-, and $x_3$-directions occur in the upper and lower parts of the cell walls and the maximal displacements in the negative $x_1$- and $x_2$-directions occur in the side walls.

In the case where all four pairs of cells are shifted relative to each other, for configurations (C1), (C2), and (C3) and boundary condition (BC1) the locations where the maximal values of the displacement occur are similar to the base case, but for configuration (C4) the maximal values of the displacement in the $x_3$-direction occur in the lower and upper parts of the cell walls; see Fig. 11.

For the geometry without a shift in the positions of the cells and boundary condition (BC1), the distribution of the maximal displacements is similar to the base case, in that for the configurations (C1), (C2), and (C4) the maximal displacements in the $x_1$- and $x_2$-directions occur in the upper and lower parts of the cell walls, and the maximal displacements in these directions for configuration (C3) occur on the side walls, and the maximal displacement in the $x_3$-direction occurs on the plane $x_3=x_{3,\mathrm{max}}$.

### Discussion of Results of Numerical Simulations

The data in Tables 4, 5, 6, 7, 8, 9 and 10 tell us several things about the impact of the presence and orientation of the cellulose microfibrils in the upper and lower parts and in the sides of the cell walls on the elastic deformation of the plant cell walls and tissues. Here a few main results are highlighted by emphazising certain data: (i) italic values contain results representing the main impact of the orientation of the microfibrils in the upper and lower parts of the cell wall; (ii) data in bold highlight the impact of applied forces and of the microstructure in the side walls on the elongation of the cells; (iii) data in bold italic indicate the effect of the microscopic structure (orientation of microfibrils in the side walls of the plant cell walls and distribution of cells in the tissue) on the strain; and (iv) with underline, we mark results that are unique to specific microfibril orientations, boundary conditions and the distribution of cells in the plant tissue.

In the case where the microfibrils in the side walls are oriented perpendicular to the cell axis, the presence and orientation of the cellulose microfibrils in the upper and lower parts of the cell wall have little effect on the expansion of the cells in the $x_3$-direction, as can be seen from looking at the columns corresponding to the maximal positive values of the displacement in the $x_3$-direction in Tables 4 and 8. The cell walls are able to expand more in the directions perpendicular to the direction of the microfibrils since the microfibrils are much stiffer than the cell wall matrix and middle lamella, and changing the microfibril orientation within the $x_1x_2$-plane has little impact on the displacement in the $x_3$-direction. However, the expansion in the $x_1$ and $x_2$-directions is affected. In particular, for (BC1) when the microfibrils are arranged in the configuration (C3) or (C3$^\prime$), the displacements in the positive $x_1$ and $x_2$-directions are approximately 2 / 3 of those for the configurations (C1) and (C2) and less than 1 / 3 of those for configuration (C4). In configurations (C3) and (C3$^\prime$), the microfibrils are oriented in both the $x_1$- and $x_2$-directions within the upper and lower parts of the cell walls and it is expected that for this configuration there would be less expansion in both directions. Notice that the difference between the maximal displacements of the plant cell walls for the configurations (C3) and (C3$^\prime$), respectively, is small. For boundary condition (BC2) a moderate difference in the maximal displacements between the configurations (C1), (C2), (C4) and (C3), (C3$^\prime$) is observed; see Table 6. In the case of boundary condition (BC3), only a noticeable difference in the maximal displacement in the positive $x_2$-direction for configurations (C1)–(C4) is observed; see Table 7. Also for boundary condition (BC3) and configuration (C2), we have a nonzero displacement in the negative $x_3$-direction in the lower parts of the cell walls (marked with underline in Table 7). This is related to the difference in the microfibril orientation in the upper and lower parts in configuration (C2) and to the difference in the turgor pressure in the neighbouring cells in boundary condition (B3). The difference in the maximal displacements for different microfibril configurations in the upper and lower parts of the cell walls for the boundary conditions (BC2) and (BC3) was less noticeable in the case of a symmetric distribution of cells without a shift in the position of the cells along the $x_3$-axis relative to each other (data not shown).

In the case where all four pairs of cells are shifted relative to each other, the noticeable difference to the base case is that for configuration (C4) the maximal values of the displacement in the $x_3$-direction occur in the lower and upper parts of the cell wall; see Fig. 11 and Table 9 (marked with underline). The fact that the maximal values for the displacement in the $x_3$-direction occur on the upper and lower parts of the cell walls is related to the large size of the corresponding upper cell and is not related to the fact that a zero normal displacement at $x_1=0$ and $x_2=0$ is prescribed (the same effect was observed when a zero normal displacement at $x_1=x_{1, \mathrm max}$ and $x_2=x_{2, \mathrm max}$ was imposed instead, date not shown). Also, for the domain with all four pairs of cells shifted, we have higher maximal values for the strain and the displacement (especially in the negative $x_1$- and $x_2$-directions) than in the base case; see Tables 4 and 9. This can be explained by the fact that the upper and lower parts of the cell walls are not equilibrated by the upper and lower parts from the neighbouring cells and larger deformations and localized displacements of the side walls near the upper and lower parts of the cell walls are possible.

If the side walls comprise layers of the microfibrils rotated through the thickness of the cell wall, the maximal displacement in the $x_3$-direction is reduced by a factor of 14 compared to the case where the microfibrils are orthogonal to the $x_3$-axis, while a slight increase of the values of the displacements in the $x_1$- and $x_2$-directions for configuration (C1), (C2), and (C4) and a slight decrease for configuration (C3) are observed; see Tables 4 and 5. The orientation of microfibrils in the upper and lower parts of the cell walls has a similar impact as in the case where the microfibrils in the side walls are orthogonal to the $x_3$-axis. Comparing Tables 4 and 5, we notice that while the displacements in the $x_3$-direction in the rotated case are smaller than the displacements in the parallel microfibril case, the strain $\mathbf{e}_{33}$ is much higher in the rotated case. This indicates that the displacements are more concentrated in particular locations in the rotated case.

**Fig. 12:**
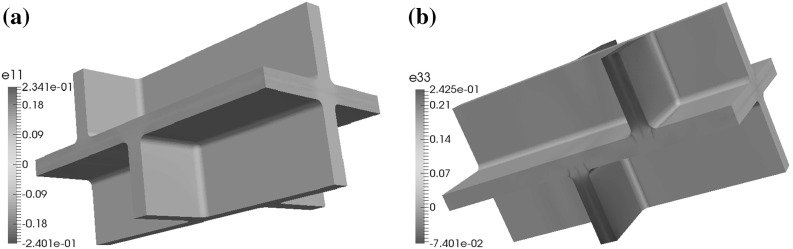
A depiction of **a** the $\mathbf{e}_{11}$ component of the strain tensor for boundary condition (BC1$^\prime$) and configuration (C4) and **b** the $\mathbf{e}_{33}$ component of the strain tensor for boundary condition (BC1$^\prime$) and configuration (C1), in the case where the geometry is as shown in Fig. 2 and the microfibrils in the side walls are orthogonal to the $x_3$-axis

Different configurations of microfibrils in the upper and lower parts of the cell walls do not induce large variations in the maximal and minimal values of the divergence of the displacement or the diagonal components of the strain tensor, see Tables 4, 5, 6, 7 and 8 and Fig. 12, apart from the minimal values for $\mathbf{e}_{33}$ which are much larger for the configuration (C2), possibly due to the orientation of microfibrils that permit larger deformations in the negative $x_3$-direction in the lower parts than in the upper parts of the cells. (Notice that for configurations (C1), (C3), (C3$^\prime$), and (C4), we have a symmetry in the microfibril distribution in the upper and lower parts.) Also in the case of a tissue with a symmetric distribution of cells for configuration (C3) we have smaller maximal values for $\mathbf{e}_{11}$ and $\mathbf{e}_{22}$ and larger minimal values for $\mathbf{e}_{11}$, $\mathbf{e}_{22}$ and $\mathbf{e}_{33}$ than for the configurations (C1), (C2), and (C4); see Table 10. This difference is smaller in the cases of the geometries with a shifted distribution of cells. We also observe that for all three geometrical configurations considered here, for configurations (C1) and (C2) the strains $\mathbf e _{11}$ and $\mathbf e _{22}$ are larger than the strain $\mathbf e _{33}$, while for (C3) and (C4) the strain $\mathbf e _{33}$ is larger than the strains $\mathbf e _{11}$ and $\mathbf e _{22}$.

Comparing the results for boundary conditions (BC2) and (BC3) in Tables 6 and 7, we can see the effect of increasing pressures in some of the cells. First, notice that the displacements in the positive directions are larger in the case (BC3) than in the case (BC2). This is because in (BC3) the pressure in cells 2, 3, 6, and 7 is greater than in the (BC2) case. Also notice that in the case (BC3) the displacement in the positive $x_1$-direction is greater than the displacement in the positive $x_2$- and $x_3$-directions, which is caused by the position of the cells with the larger pressure. Namely, there is a pressure difference between the cells that are aligned in the $x_1$-direction. Even though there are no tensile tractions on the sides of the domain for boundary conditions (BC2), due to the microscopic structure of the side walls, defined by the microfibrils orthogonal to the $x_3$-axis, $\mathbf e _{33}$ is larger than $\mathbf e _{11}$ and $\mathbf e _{22}$; see Table 6. Also, the difference in the pressure in the neighbouring cells in boundary condition (BC3) induces extra strains; compare Tables 6 and 7.

For a larger tensile traction, i.e. $f=3.426 p_{\circ ,1}$ with $p_{\circ ,1}=0.209$ MPa, we observe the same impact of the orientation of the microfibrils in the upper and lower parts of the cell walls as in the base case. We find that a 16.5 % increase in the tensile traction results in an approximately 13–16 % increase in the maximal and minimal values of the divergence of the displacement and the diagonal components of the strain tensor and in the maximal values of the displacements in the positive and negative $x_1$-, $x_2$-, and $x_3$-directions (data not shown). The increase in the turgor pressure from $p_{\circ ,1}=0.209$ MPa to $p_{\circ ,2}=0.3$ MPa with the same value for the tensile traction boundary condition, i.e. $f=2.047p_{\circ ,2}$, also results in a slight ($\sim$5–10 %) increase in the corresponding values for the displacements, the diagonal components of the strain tensor and the divergence of the displacement; compare Tables 4 and 8. The impact of the orientation of the microfibrils in the upper and lower parts of the cell walls is similar to the base case.

For a geometry with shorter cells (i.e. 1/2 of the length) and without a shift in the position of cells, the only significant difference is that the displacement in the $x_3$-direction for the larger cells is twice the displacement for the smaller cells (data not showed). This is in accord with Hooke’s law, which tells us that the elongation of an elastic bar under an applied load is a linear function of the length of the bar.

## Discussion and Conclusion

Our results indicate that in the case of (i) directed tensile forces applied to plant cells and tissues, (ii) tissue tension created by different values of turgor pressure in the neighbouring cells, and/or (iii) the staggered distribution of cells in plant tissues, the orientation of the microfibrils in the lower and upper parts of the cell walls plays a role and may be important for the expansion and development of plant tissues; see the italic values in Tables 4, 5, 7, 8, 9 and 10. The orientation of the microfibrils in the upper and lower parts of plant cell walls has a very small effect on the elongation of the cells, but it influences their radial expansion. Also, the qualitative impact of the orientation of microfibrils in the upper and lower parts of cell walls on the deformation of plant tissues does not depend on the actual values for the tensile forces and turgor pressure, and the increase in tensile traction or turgor pressure results only in the corresponding increase in the maximal values for the displacements.

The staggered distribution of cells in a plant tissue induces a different tissue tension than in the case of a tissue without a shift in the positions of neighbouring cells. Also, the staggered distribution of cells allows for larger deformations and larger values of the diagonal components of the strain tensor. This can be explained by the fact that the upper and lower parts of the cell walls are not equilibrated by the upper and lower parts from the neighbouring cells. The higher maximal values of the displacements in the negative $x_1$- and $x_2$-directions for the geometries with the staggered distribution of cells, compared to the geometry without a shift in the positions of the neighbouring cells, constitute a noticeable difference between three geometries considered here. Also, for the geometry without a shift in the positions of neighbouring cells along the $x_3$-axis, we have a uniform deformation of the side walls, whereas in the two other cases we observe nonuniform patterns in the displacement and larger values of the displacement occur near the lower and upper parts of the cell walls. The ability of larger and nonuniform deformations can be favourable for plants and may be one of the explanations for the staggered distribution of cells in plant tissues.

The orientation of microfibrils in the side walls has a strong impact on the deformation of the plant cell walls and tissues. If the microscopic structure of the side walls is given by the layers of the microfibrils rotated through the thickness of the cell wall, the maximal displacement in the $x_3$-direction is reduced by a factor of 14 compared to the case where the microfibrils are orthogonal to the $x_3$-axis. The higher values for the strain $\mathbf{e}_{33}$ in the case of rotated microfibrils, compared to the case where the microfibrils are orthogonal to the $x_3$-axis, constitute a nonintuitive result; see Tables 4, 5 and 9 (the corresponding values are in bold italic). These large values for the strain $\mathbf{e}_{33}$ may be important for some stress-related signalling processes, e.g. related to the reorientation of microtubules (Hamant et al. 2008). Comparing Tables 4 and 7, we also see that the presence of the tensile traction boundary condition causes the displacements in the positive directions to increase by an order of magnitude.

We also obtain that the different pressures in neighbouring cells, which can be observed during the growth process, influence the direction of the maximal displacement; see Table 7 (here the maximal displacement in the $x_1$-direction is due to pressure distributions).

**Table 11:** The relative displacement (RD) is defined as the maximal deformed length in the $x_3$-direction divided by the initial length in the $x_3$-direction

RD	(BC1) parallel MF	(BC1) no shift	(BC1) 4 shifts	(BC1) rotated MF	(BC2)	(BC3)
(C1)	1.06497	1.06396	1.06984	1.00456	1.00683	1.00816
(C2)	1.06452	1.06391		1.00462	1.00671	1.00823
(C3)	1.06234	1.06294	1.06680	1.00379	1.00672	1.00831
(C4)	1.06411	1.06320		1.00497	1.00696	1.00811

Using the fact that for most cases considered here (besides the case (BC1), (C4) for the geometry where all four pairs of cells are shifted relative to each other) the maximal values for the displacement in the positive $x_3$-direction occur on the plane $x_3=x_{3,\mathrm{max}}$, we can calculate the relative displacement (RD) in the $x_3$-direction, defined by the maximal deformed length in the $x_3$-direction divided by the initial length in the $x_3$-direction. This quantity can be related to the measurements of the changes in the length (extension or compression) of strips or cylinders of an outer or inner tissue, respectively, due to the elimination of tissue tension by separating them from the plant hypocotyl (Hejnowicz and Sievers 1995). In our numerical simulations, we used the same tension at the boundary of the plant tissue as in the experiments. The relative changes in the length obtained from our mathematical model range between 0.38 and 6.98 %, see Table 11, and are in relatively good agreement with the experimental results ranging between 0.3 and 4.99 %; see Table 1 in Hejnowicz and Sievers (1995). The small relative changes in the length correspond to the case where no tensile forces were applied and to the case where the microstructure of the side cell walls was given by layers of rotated microfibrils. Notice that the comparison between the results obtained from our mathematical model and the experimental results must be taken with the caveat that the mathematical model is defined on the scale of a few cells, whereas the experiments are performed on the tissue level. However the good agreement between the model and experiments provides a basis for further analysis of the mechanical properties of plant tissues using our multiscale mathematical model. For a more accurate comparison to the tissue level experiments, our model can also be generalized to the tissue level, which will be the subject of future research.

Also, in our model we assumed that the microfibrils on the sides of the cell walls are arranged in fixed rings around the cells without considering possible sliding of the microfibrils during the expansion. The effect of the sliding of the microfibrils on the deformation of plant cells and tissues in combination with different arrangements of microfibrils in the upper and lower parts of the cell walls will be analysed in future studies. Moreover, we will consider the relation between the rotated macroscopic elasticity tensor, considered here to define the macroscopic elastic properties of the side walls comprising microfibrils rotated across the thickness of the cell walls, and the macroscopic elasticity tensor for a plywood-like microstructure obtained by applying the locally periodic homogenization (Ptashnyk 2015).
